# Effect of early mobilization combined with early nutrition on acquired weakness in critically ill patients (EMAS): A dual-center, randomized controlled trial

**DOI:** 10.1371/journal.pone.0268599

**Published:** 2022-05-26

**Authors:** Wendie Zhou, Lili Yu, Yuying Fan, Baisheng Shi, Xiaohui Wang, Tianling Chen, Haixia Yu, Jie Liu, Xizhen Wang, Caihong Liu, Huijia Zheng

**Affiliations:** 1 Clinical Nursing Teaching Department, Second Affiliated Hospital of Harbin Medical University, Harbin, Heilongjiang, China; 2 School of Nursing, Harbin Medical University, Harbin, Heilongjiang, China; 3 Nursing Department, Heilongjiang Academy of Traditional Chinese Medicine, Harbin, Heilongjiang, China; 4 Department of Rehabilitation, Second Affiliated Hospital of Harbin Medical University, Harbin, Heilongjiang, China; 5 Department of Intensive Care Unit, Second Affiliated Hospital of Harbin Medical University, Harbin, Heilongjiang, China; 6 Department of Intensive Care Unit, The First Hospital of Qiqihar, Qiqihar, Heilongjiang, China; University of Texas Medical Branch at Galveston, UNITED STATES

## Abstract

**Aim:**

The study aimed to investigate the effect of early mobilization combined with early nutrition (EMN) on intensive care unit-acquired weakness (ICU-AW) in intensive care unit (ICU) settings compared with early mobilization (EM) or routine care.

**Methods:**

A prospective, dual-center, randomized controlled trial was conducted. The control group underwent standard care without a pre-established routine for mobilization and nutrition. The EM group underwent early, individualized, progressive mobilization within 24 h of ICU admission. The EMN group underwent early mobilization, similar to the EM group plus guideline-based early nutrition (within 48 h of ICU admission). The primary outcome was the occurrence of ICU-AW at discharge from the ICU. Secondary outcomes included muscle strength, functional independence, organ failure, nutritional status, duration of mechanical ventilation (MV), length of ICU stay, and ICU mortality at ICU discharge.

**Results:**

A total of 150 patients were enrolled and equally distributed into the three groups. Patients undergoing routine care only were more susceptible to ICU-AW upon ICU discharge than those in the EM or EMN groups (16% vs. 2%; *p* = 0.014 for both), and had a lower Barthel Index than others (control vs. EM/EMN: 57.5 vs 70.0; *p* = 0.022). The EMN group had improved muscle strength (*p* = 0.028) and better nutritional status than the control group (*p* = 0.031). Both interventions were associated with a lower ICU-AW (EM vs. control: *p* = 0.027, OR [95% CI] = 0.066 [0.006–0.739]; EMN vs. control: *p* = 0.016, OR [95% CI] = 0.065 [0.007–0.607]).

**Conclusion:**

EM and EMN had positive effects. There was little difference between the effects of EM and EMN, except for muscle strength improvement. Both EM and EMN may lead to a lower occurrence of ICU-AW and better functional independence than standard care. EMN might benefit nutritional status more than usual care and promote improvement in muscle strength.

## Introduction

Intensive care unit-acquired weakness (ICU-AW) is a severe neuromuscular complication in critically ill patients, with a global incidence of 25–31% among the critically ill [1]. ICU-AW can affect the respiratory and limb muscles, leading to prolonged mechanical ventilation (MV), extended intensive care unit (ICU) stay, higher hospital costs, increased mortality, and decreased quality of life [2–4]. It can take weeks to months to achieve functional recovery from ICU-AW, with most severe cases unable to regain their level of function before the onset of critical illness [4, 5]. Although some risk factors for ICU-AW have been identified, its pathogenesis and etiology remain uncertain, and there are no specific drugs or targeted treatments to offset these changes.

Early mobilization (EM) is a promising potential intervention to combat ICU-AW, but it still requires further exploration [6–10]. Mobilization could possibly shorten the continuous immobilization prevalent in the ICU and encourage muscle loading, contributing to both the muscle protein synthesis pathway stimulation and suppression of catabolism [5]. Additionally, early initiation of nutritional intake can suppress excessive immune responses, inhibit tissue damage caused by inflammation, reduce secondary infection, and advance intestinal function recovery, thus preventing ICU-AW [11, 12].

Recently, it has been suggested that a combination of adequate nutrient intake and exercise may improve muscle protein anabolism, decrease degradation, preserve muscle mass, and enhance physical performance in critically ill patients [13, 14]. In addition, a combination of both might be more effective than nutritional intervention or rehabilitation therapy alone [15]. Nutritional support for critically ill patients can arouse their enthusiasm to participate in activities and exercise, and joint early mobilization and nutritional intervention may reduce mortality, decrease muscle mass loss, prevent ICU-AW, reduce the damage of related body functions, and accelerate the speed of disease recovery [13, 16, 17]. However, few studies have explored the effect of early mobilization combined with early nutrition (EMN) on ICU-AW or compared the combined effects with one of these interventions alone.

The primary aim of this early mobilization combined with early nutrition on acquired weakness in critically ill patients (EMAS) trial was to investigate the combined effects of early mobilization and early nutrition (early start of nutritional intake) on ICU-AW in adult ICU patients. We hypothesized that early mobilization combined with early nutrition would prevent ICU-AW more than early mobilization alone or standard care in patients admitted to the ICU.

## Materials and methods

### Study design

The EMAS was a dual-center, three-group, randomized controlled trial (RCT) conducted at four ICUs (surgical ICU, medical ICU, emergency ICU, and cardiac surgical ICU) of the Second Affiliated Hospital of Harbin Medical University and one ICU (general ICU) of the First Hospital of Qiqihar. The First Affiliated Hospital of Jiamusi University, which was planned to be one of the trial sites, was replaced by the First Hospital of Qiqihar owing to the Coronavirus disease 2019 (COVID-19) pandemic. Ethical approval was provided by the Ethics Committee of the Second Affiliated Hospital of Harbin Medical University, Harbin, China (KY 2020–012). The protocol was registered at Chictr.org.cn (ChiCTR2000033482). The full details of the trial design have been published [3]. The manuscript was prepared based on the CONSORT guidelines for multi-arm parallel-group randomized trials [18].

### Randomization and blinding

A blinded external researcher prepared the randomization sequence using Microsoft Office Excel software. The details of the study procedure are described in a previously published protocol [3]. Participants were randomized in a 1:1:1 ratio to the standard care (control), EM (intervention), or EMN (intervention) groups as soon as eligibility was confirmed after screening.

The enrolled patients, researchers, and clinical staff could not be blinded to the group allocation during the implementation of the EM intervention. Physiotherapists assessing the Medical Research Council (MRC) sum score and the Barthel Index (BI) could not be blinded. However, to minimize bias, each assessment was performed by two physiotherapists individually; if their results varied, a third therapist was required. The other outcomes were collected by blinded researchers. The group allocation was concealed from recruiters who were responsible for assessing and collecting the baseline characteristics of eligible patients. The data analyst performing the analysis of the outcomes was also blinded to the allocation. Patients in the EM and EMN groups were blinded to whether they had received an early nutrition protocol.

### Study participants

Patients admitted to the ICU, enrolled prospectively, were screened consecutively for inclusion if they were: 1) ≥18 years of age; 2) were admitted to the ICU for the first time; 3) had an expected ICU stay ≥72 h; 4) were conscious enough within the subsequent 24 h to respond to at least three of the following orders: “open and/or close your eyes,” “look at me,” “put out your tongue,” “nod your head,” and “raise your eyebrows;” and 5) had a BI ≥ 70 at 2 weeks before ICU admission. Patients who met the following criteria were excluded: 1) pregnancy; 2) deformity, paralysis, fracture, or surgery of limbs; 3) pre-existing primary systemic neuromuscular disease that affects muscle strength (e.g., Guillain Barre, myasthenia gravis, amyotrophic lateral sclerosis); 4) intracranial or spinal processes affecting motor function; 5) gastrointestinal surgery within 1 month; 6) no expectation of any nutritional intake within the subsequent 48 h; and 7) terminal cancer, expected death, or extremely poor prognosis. Both mechanically ventilated and nonmechanically ventilated patients were included. As soon as the patients met the criteria, they or their legally authorized representatives would be contacted to obtain their informed consent. Patients and/or legal representatives who refused to participate were excluded.

### Interventions

Except the protocols of mobilization and nutritional support, other treatments and nursing care were the same for all. Apart from the EMN group, the other two groups (control and EM groups) received conventional nutritional support. The feeding speed and nutrient composition were consistent among the three groups. Enteral Nutritional Suspension produced by NUTRICIA (Wuxi, Jiangsu, China) was used as the enteral nutrition solution. Parenteral nutrition (PN) solutions were fat emulsion, amino acids (17), and glucose (11%) injections made by Fresenius Kabi AB (Uppsala, Sweden). Nutritional targets were measured using the weight-based equation, 20–25 kcal/kg/d, and protein equivalents of 1.3 g/kg/d were provided progressively [16]. All the patients who required enteral nutrition (EN) were administered continuous feeding. The intervention and outcome measurement ceased at ICU discharge, considering the lower risk of muscle wasting, physical impairment, and weakness after resolution of critical illness and numerous interfering factors beyond control after ICU discharge [13]. The interventions were implemented by the researchers and trained personnel of the research team. The members of the research team included ICU physicians, physiotherapists, dietitians, responsible nurses, head nurses, and researchers. The physiotherapists performing routine rehabilitation were the same therapists performing EM.

#### Control group

The control group underwent standard ICU rehabilitation and nutritional support only, as follows:

1. Routine rehabilitation exercise: There were no physiotherapy protocols in the units or physiotherapists available at all times. The physiotherapist was invited to perform rehabilitation exercise therapy for certain patients, according to the ICU physician’s order. The conventional rehabilitation method was decided by a physiotherapist based on experience, involving mostly passive mobilization, once daily, for approximately 15 min/session. The initiation time was determined by the ICU physician based on their experience. Whenever physiotherapy was ordered by the physician, routine rehabilitation was commenced the next day.
2. Routine nutritional support: There is no pre-established nutrition protocol in the units. The commencement time and route of administration of nutritional support were determined as ordered by the physician based on experience. The feeding tube was kept in place. The head of the bed was raised to 30–45 degrees. Gastrointestinal syndromes (e.g., diarrhea and abdominal distension) were monitored. The feeding speed was adjusted according to the patient’s condition and reaction. Feeding was paused during exercise to prevent aspiration. Blood glucose and electrolyte levels were measured at least every 4 h. The blood glucose level was maintained at 6–10 mmol/L, with intravenous insulin administered if necessary.

#### Early mobilization group

The EM group underwent the early mobilization intervention in addition to standard ICU care. Physiotherapists, ICU physicians, responsible nurses, and researchers collaborated on the implementation of EM within 24 h of ICU admission of the patient, twice daily, 20–30 min/session, until ICU discharge. The EM was designed based on Orem’s theoretical framework [19]. The mobilization mode that the patients received was determined by their functional independence, measured daily by BI [20]. Patients with different BI were assigned to the corresponding nursing systems, thus receiving individualized mobilization in accordance with their own functional independence. The content of the EM protocol was based on literature [9, 21] and expert consultation. Some details of the EM intervention, such as the exercise duration and the number of times certain movements were performed, were adjusted after the pilot study and before the initiation of the formal trial. A walking task was also added to mode 3. The details of the EM intervention are presented in the S1 File.

#### Early mobilization combined with early nutrition group

Early nutrition intervention, apart from standard care and EM intervention (the same as received by the EM group), was applied in the EMN group with the cooperation of ICU physicians, the dietician, responsible nurses, and researchers. The early nutrition protocol was initiated within 48 h of ICU admission. The route of administration of nutritional support was determined based on the patient’s nutritional risk assessed using the Nutrition Risk Screening (NRS) 2002, nutritional status measured using the subjective global assessment (SGA), and whether there was a contraindication for EN. It was designed based on the 2019 European Society for Clinical Nutrition and Metabolism (ESPEN) guideline and dietician counsel. More details on early nutritional intervention are provided in the protocol [3]. The only difference between the early nutrition protocol and the standard ICU nutritional support was the initiation time and the method of choosing the feeding route, while other aspects (including feeding speed, ingredients, and energy consumption calculation, etc.) were the same. Regarding the feeding initiation time, patients receiving early nutrition protocol commenced nutritional intake within 48 h after ICU admission, while the initiation time for those who followed routine nutrition support was decided based on the doctors’ experience. In terms of the method of choosing feeding routes, that of the EMN group was decided following the ESPEN guideline as described above, while that of the control and EM groups was based on experience.

### Outcome measures

The primary outcome was the occurrence of ICU-AW at ICU discharge, defined as an MRC sum score of < 48 [22].

The secondary outcomes included overall muscle strength evaluated by the MRC sum score, functional independence by BI, organ failure by sequential organ failure assessment (SOFA), nutritional status by SGA, duration of MV, length of ICU stay, and ICU mortality at ICU discharge.

The MRC sum score [22] is used to evaluate the muscle strength of six pairs of muscle groups. The total score ranges from 0 (quadriplegia) to 60 (normal muscle strength). A total score of less than 48 is commonly used for the diagnosis of ICU-AW. Although the MRC sum score is the gold standard, the results can vary owing to the subjective nature of the manual muscle test.

BI assessment [20] includes 10 different activities of daily living. The higher the score, the better the functional independence and self-care abilities.

SOFA score [23] may be associated with loss of muscle mass [24]. The outcome in this study was ΔSOFA score, representing the change of SOFA score after the trial (ΔSOFA score = total maximum SOFA (TMS) score − total SOFA score at ICU admission), which accounts for the influence of SOFA score during ICU admission.

Nutritional status was evaluated using the SGA [25], which is recommended by the American Society for Parenteral and Enteral Nutrition (ASPEN) and ESPEN [16] and can be used for critically ill patients [26].

All of the above tools have been shown to be valid and reliable in the ICU/acute setting. The outcomes measured at enrolment were evaluated by recruiters (responsible nurses). Functional outcome assessments (MRC sum score, BI) during ICU stay and at ICU discharge were performed by two physiotherapists individually. Other outcomes collected during ICU stay and at ICU discharge were assessed by the researchers. Details on the collection of outcomes can be found in the S1 File. Data for the outcomes that did not require additional manual assessment (MRC sum score and BI, which were not assessed or recorded routinely in the ICU) were obtained from electronic medical records of the patients and face-to-face conversations with the patients or their responsible proxies.

### Sample size and statistical analysis

The sample size was calculated using PASS (version 11.0.7) software, with a two-sided 5% level of significance and a power of 90%; a chi-square test with two degrees of freedom, allowing for 10% sample loss, was used in determining the sample size. The estimated effect size was based on the incidence of ICU-AW at ICU discharge (control group, 51.9%; EMN group, 33.1%) reported in a meta-analysis by Zang et al. [27] and Chen et al. [28] (15.9%). Considering the 10% dropout rate (15 participants) during the trial, it was estimated that at least 147 participants were required to detect a significant between-group difference in the primary outcome (incidence of ICU-AW).

Both intention-to-treat (ITT) and per-protocol (PP) analyses were conducted for the sensitivity analyses. The normality of the variables was assessed using the Kolmogorov–Smirnov test. Continuous data are expressed as mean ± standard deviation (SD) (normal variables) or medians (P_25_–P_75_) (non-normal variables), and categorical data are presented as frequencies (%). The 95% confidence interval (CI) for incidence rate was calculated by Clopper–Pearson method, expressed as percentages. Normal variables among the three groups were compared by one-way ANOVA, non-normal variables and ranked data with nonparametric Kruskal–Wallis test, and categorical data (except for ranked data; e.g., incidence rates of ICU-AW across the three groups) as well as their pairwise comparisons (preconditioned by select case) with chi-squared or Fisher’s exact tests. Data with statistical differences revealed by the Kruskal–Wallis test were further compared pairwise with *p-values* adjusted by Holm–Bonferroni correction. Intragroup comparisons of data before and after the intervention were performed using the nonparametric Wilcoxon signed-rank test (related samples).

A logistic regression model was used to evaluate the effect of EM and EMN on the occurrence of ICU-AW adjusted for covariates, including Acute Physiology and Chronic Health Evaluation (APACHE) II score and gastrointestinal/hepatic disorder. The selection of covariates was based on the change-in-estimate method, which is the most popular data-driven approach for selecting covariates [29]. According to this method, if the removal of one covariate leads to a less than 10% change in odds ratio (OR), then this covariate should not be included in the final model [30].

Statistical analyses were conducted by an independent statistician using SPSS Statistics software (version 24.0) and GraphPad Prism software (version 9.1.1), using two-tailed tests with *p-values* below 5% indicating statistical significance.

## Results

**Fig 1:**
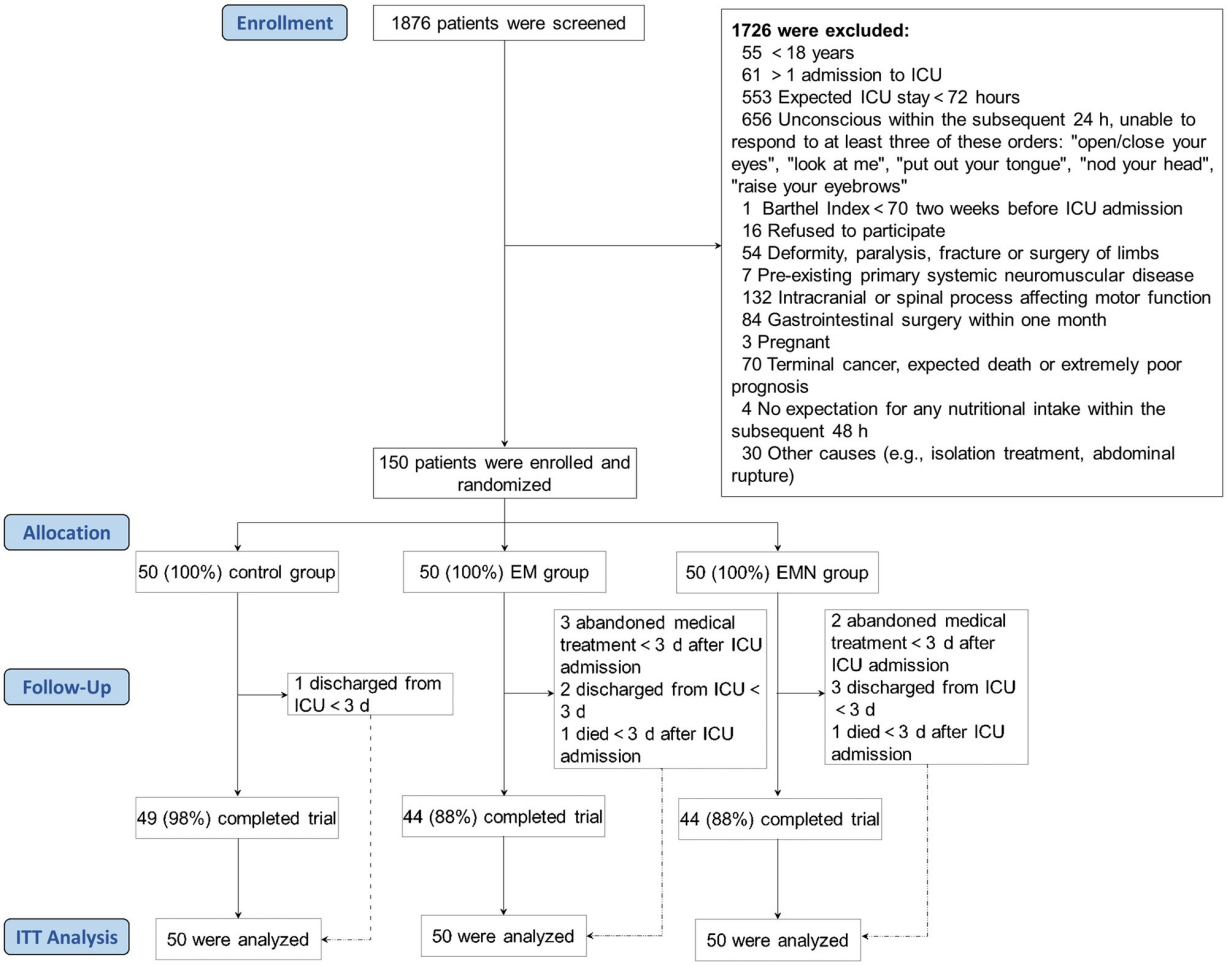
Flowchart of the trial. EM, early mobilization; EMN, early mobilization combined with early nutrition.

From September 7, 2020 to May 22, 2021, 1876 patients were admitted consecutively to the ICUs and were screened for eligibility in the first 24 h of their ICU stay; 150 eligible patients were enrolled and randomized into the three groups, 50 in each group. Thirteen patients did not complete the protocol due to death, ICU discharge, or abandoning medical treatment in less than 3 days of ICU admission. The results of ITT and PP analyses did not differ considerably. The ITT paradigm can avoid overestimation of the effect, and thus is a more conservative approach in a clinical trial [31]. According to the ITT principle, only when no treatment is implemented or no data is available after randomization should the trial not adopt ITT [31]. Therefore, ITT results were adopted in this trial. The study flow is presented in Fig 1, and the baseline variables are shown in Table 1. No significant differences were found among the three groups in baseline variables.

**Table 1:** Baseline demographic and clinical characteristics.

Variables	Control Group (n = 50)	EM Group (n = 50)	EMN Group (n = 50)	*p*
Gender, female, no (%)	20 (40)	24 (48)	26 (52)	0.472
Age (yr), mean ± SD	57.3 ± 13.7	57.0 ± 15.3	58.7 ± 14.9	0.825
BMI (kg/m^2^), mean ± SD	23.9 ± 4.0	24.3 ± 3.6	23.2 ± 4.4	0.405
APACHE II score, mean ± SD	14.0 ± 6.3	13.9 ± 5.1	16.0 ± 5.2	0.102
MV, n (%)	14 (28)	13 (26)	18 (36)	0.513
Education level, no (%)				0.579
Primary school or less	14 (28)	14 (28)	16 (32)	0.879
Middle school	22 (44)	14 (28)	17 (34)	0.239
High school	9 (18)	12 (24)	8 (16)	0.574
College or above	5 (10)	10 (10)	9 (18)	0.353
Diagnosis, n (%)				0.059
Renal failure	9 (18)	3 (6)	13 (26)	0.026
Gastrointestinal/hepatic disorder	6 (12)	11 (22)	4 (8)	0.115
Respiratory disorder	9 (18)	9 (18)	11 (22)	0.843
Acute pancreatitis	4 (8)	6 (12)	2 (4)	0.394
Cardiac disorder	9 (18)	2 (4)	5 (10)	0.075
Infection	2 (4)	4 (8)	6 (12)	0.394
Others ^a^	11 (22)	15 (30)	9 (18)	0.352
High risk factors of ICU-AW, n (%)				0.378
Diabetes	6 (12)	10 (20)	7 (14)	0.513
Infection	11 (22)	13 (26)	14 (28)	0.781
Post-surgery	7 (14)	2 (4)	2 (4)	0.113
None	16 (32)	21 (42)	16 (32)	0.482
Two factors combined	9 (18)	4 (8)	9 (18)	0.264
Three factors combined	1 (2)	0 (0)	2 (4)	0.773
Barthel Index (two weeks before ICU admission), median (P_25_-P_75_)	100 (100–100)	100 (98.75–100)	100 (95–100)	0.228
NRS 2002 score, median (P_25_-P_75_)	4 (3–6)	4 (3–6)	5 (4–6)	0.127
SOFA score, median (P_25_-P_75_)	6 (4–8)	6 (3.8–7.0)	6 (4.0–8.3)	0.908
Medical insurance (no/yes), n (%)	20 (40)/30 (60)	32 (64)/18 (36)	27 (54)/23 (46)	0.054

APACHE II, Acute Physiology and Chronic Health Evaluation II; BMI, Body Mass Index; EM, early mobilization; EMN, early mobilization combined with early nutrition; ICU-AW, intensive care unit-acquired weakness; MV, mechanical ventilation; NRS 2002, nutrition risk screening 2002; SD, standard deviation; SOFA, sequential organ failure assessment.

^a^ Others include: poisoning, electrolyte disturbances, systemic lupus erythematosus, etc.

### Primary outcome

#### Occurrence of ICU-AW

After the intervention, there was a significant difference in the occurrence of ICU-AW between the three groups (control group: 16%, 95% CI 7.2–29. 1%; EM group: 2%, 95% CI 0.1–10.6%; EMN group: 2%, 95% CI 0.1–10.6%) at ICU discharge (*p* = 0.005) (Table 2). Participants in the control group were more likely to have ICU-AW at endpoint than those in the EM group (*p* = 0.014) or EMN group (*p* = 0.014), with the figures for the EM and EMN groups (2% for both) seven times less than those in the control group (16%).

**Table 2:** Comparison among groups for outcomes assessed at ICU discharge only.

Variables	Control Group (n = 50)	EM Group (n = 50)	EMN Group (n = 50)	*p*
ICU-AW, n (%) (95% CI (%))	8 (16) (7.2–29. 1)	1 (2) (0.1–10.6)	1 (2) (0.1–10.6)	0.005^a^
ΔSOFA score^b^, median (P_25_-P_75_)	0 (0–3)	0 (0–3)	0 (0–2)	0.614
Length of ICU stay (d), median (P_25_-P_75_)	4.1 (3.2–6.0)	4.5 (3.0–7.7)	3.4 (3.0–4.9)	0.040
Duration of MV (h), median (P_25_-P_75_)	0 (0–42.2)	0 (0–61.9)	0 (0–56.5)	0.753
Death, n (%)	2 (4)	3 (6)	2 (4)	1.000

CI, confidence interval; EM, early mobilization; EMN, early mobilization combined with early nutrition; ICU, intensive care unit; ICU-AW, intensive care unit-acquired weakness; MV, mechanical ventilation; SOFA, sequential organ failure assessment.

^a^ Difference between control group and EM group (*p* = 0.014), and between control group and EMN group (*p* = 0.014)

^b^ ΔSOFA score = total maximum SOFA score − total SOFA score at ICU admission

### Secondary outcomes

#### Muscle strength

**Fig 2:**
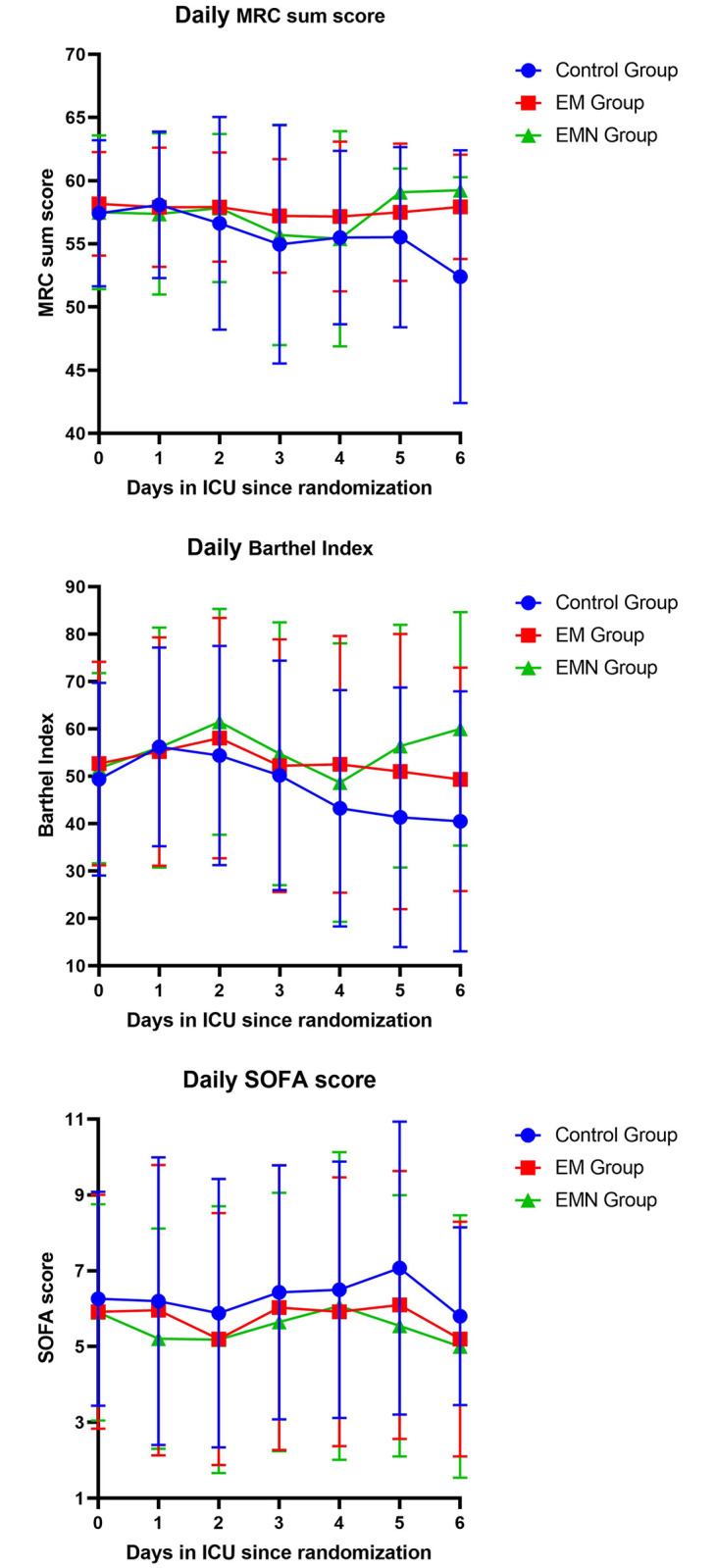
Mean daily MRC sum score, Barthel Index and SOFA score per trial day 0–6 for the three groups. Error bars are SD for means in the three groups at each time point. EM, early mobilization; EMN, early mobilization combined with early nutrition; ICU, intensive care unit; MRC, Medical Research Council; SD, standard deviation; SOFA, sequential organ failure assessment.

The MRC sum score did not differ among the groups either before or after the trial (Table 3). The score in the EMN group after the intervention was substantially higher than before the intervention (*p* = 0.028). As shown in Fig 2, from days 0 to 6 after randomization, the MRC sum scores of patients in the EM and EMN groups tended to be higher than those of the control group on most study days. The figure for the control group began to fall after day 4, while that for the EMN group started to rise to a level higher than that of the EM group, which was almost the highest over the first 4 days.

**Table 3:** Comparison among groups for outcomes assessed both before and after applying the study protocol.

Variables	Control Group (n = 50)	EM Group (n = 50)	EMN Group (n = 50)	*p*
MRC sum score, median (P_25_-P_75_)				
Before	60.0 (58.5–60.0)	60.0 (58.8–60.0)	60.0 (58.0–60.0)	0.977
After	60.0 (56.5–60.0)	60.0 (59.8–60.0)	60.0 (60.0–60.0)^a^	0.225
Barthel Index, median (P_25_-P_75_)				
Before	50.0 (35.0–65.0)	57.5 (43.8–65.0)	57.5 (35.0–66.3)	0.714
After	57.5 (38.8–70.0)	70.0 (50.0–81.3)^b^	70.0 (55.0–80.0)^b^	0.008^c^
SGA rating, n (%)				
Before				0.504
SGA A	15 (30)	16 (32)	6 (12)	0.013
SGA B	14 (28)	17 (34)	27 (54)	0.107
SGA C	21 (42)	17 (34)	17 (34)	0.083
After				0.025^d^
SGA A	16 (32)	26 (52)	26 (52)	0.230
SGA B	23 (46)	17 (34)	22 (44)	0.368
SGA C	11 (22) ^a^	7 (14)^a^	2 (4)^b^	<0.001

EM, early mobilization; EMN, early mobilization combined with early nutrition; MRC, Medical Research Council; SGA, subjective global assessment.

^a^ Different than before (*p*<0.05)

^b^ Extremely different than before (*p*<0.001)

^c^ Difference between control group and EM group (*p* = 0.022), and between control group and EMN group (*p* = 0.020)

^d^ Difference between control group and EMN group (*p* = 0.031)

#### Functional independence

BI was significantly better in the EM and EMN groups upon ICU discharge (EM/EMN group vs. control group: 70.0 vs. 57.5; *p* = 0.022) (Table 3). In addition, except for the control group (*p* = 0.586), the BI of the EM and EMN groups showed significant improvement after the intervention (*p* < 0.001 for both). The change in daily BI in the three groups was similar to that in the daily MRC sum score during the days shown (Fig 2). The BI of the EMN group remained the highest among the three groups most of the time.

#### Organ failure

The ΔSOFA score, which indicates the change in organ failure after the intervention, showed no statistical difference among the three groups (Table 2). In terms of the daily SOFA score, that of the control group was the highest throughout the first 6 days of the trial (Fig 2), while that of the EMN group was the lowest. The scores in all the groups began to decrease on day 5.

#### Nutritional status

Significant differences in SGA ratings among the three groups (*p* = 0.025) are shown in Table 3. The SGA rating in the EMN group was better than that in the control group after the trial (*p* = 0.031). The ratings of all groups showed significant improvement compared with those before the intervention (*p* = 0.040, *p* = 0.001, and *p* < 0.001 for the control, EM, and EMN groups, respectively).

#### Other secondary outcomes

We found a statistically significant difference in the length of ICU stay between the three groups (*p* = 0.040) (Table 2). The EMN group had a shorter ICU stay than the control (3.4 d vs 4.1 d) and EM (3.4 d vs 4.5 d) groups, but there was no statistical difference after multiple-comparison adjustment. No significant difference was observed in the duration of MV and ICU mortality at discharge from the ICU (Table 2).

### Analysis of predictive variables for ICU-AW

Compared to the control group, the EM group had approximately 93.4% | (6.6%–1) | (the absolute value of (OR-1)) less chance of developing ICU-AW (*p* = 0.027). The likelihood of developing ICU-AW in the EMN group was approximately 93.5% | (6.5%–1) |less than that in control group (*p* = 0.016) (Table 4).

**Table 4:** Impact of interventions on ICU-AW during ICU stay adjusted for covariates.

Variables	OR (95% CI)	*p*
APACHE II score	1.222 (1.072–1.392)	0.003
Gastrointestinal/hepatic disorder		
No	Referent	
Yes	5.001 (0.686–36.471)	0.112
Study group		
Control	Referent	
EM	0.066 (0.006–0.739)	0.027
EMN	0.065 (0.007–0.607)	0.016

APACHE II, Acute Physiology and Chronic Health Evaluation II; CI, confidence interval; EM, early mobilization; EMN, early mobilization combined with early nutrition; ICU, intensive care unit; ICU-AW, intensive care unit-acquired weakness; OR, odds ratio.

## Discussion

To the best of our knowledge, this EMAS trial is the first to investigate the effect of early nutrition combined with early mobilization on ICU-AW compared with early mobilization alone or standard care. The results from this study add to the literature on joint early nutrition and early mobilization programs, as well as early, progressive, and individualized mobilization practices, along with their benefits for the critically ill. Additionally, this trial provides evidence for the feasibility of incorporating Orem’s theoretical framework into mobilization programs for ICU patients, in an effort to achieve individualized exercise and to encourage the patients to try to move on their own in the ICU setting, realizing their crucial role in combating ICU-AW and critical illness, thus regaining mobility and autonomy.

This study revealed the benefits of EMN in ICU-AW prevention, functional independence, nutritional status, muscle strength, and muscle strength improvement compared to usual care. EM alone might also be related to a lower occurrence of ICU-AW, better functional independence, and more satisfactory muscle strength. We did not find any differences in organ failure, duration of MV in the ICU, or ICU mortality among the three groups.

The possible positive impact of EM on ICU-AW prevention and functional independence promotion found in this study are consistent with the findings of recent meta-analyses [27, 32], further proving the benefits of early, individualized, progressive mobilization strategies in combating ICU-AW and regaining self-care ability. In this trial, it was found that the addition of EM could result in an approximately 89% lower chance of developing ICU-AW compared to standard ICU care. It was also suggested by a recent RCT investigating the effect of an early progressive mobilization program that even 1% more activity in the ICU could lead to 35% more likelihood of functional independence at ICU discharge, and simply participating in this kind of program could result in 22 times better chances of good functional independence [33]. Apart from the progressive mobilization design in the EMAS trial, Orem’s theoretical framework [19] was applied to the EM intervention. This theory has not been adopted in previous mobilization programs for ICU patients. By applying Orem’s theoretical framework, we aimed to not only offer individualized mobilization catering to the patients’ mobility status but also to encourage the patients to move actively to regain their mobility and self-care ability by themselves in the ICU, rather than be passively driven by the caregivers’ orders. We explained to the participants the benefits that this form of activity might have on their mobility, functional independence, and self-care ability; relieved their concerns about safety issues; and raised their confidence regarding their ability to perform these activities despite their critical illness. This is because motivation, beliefs (especially regarding benefits and/or risks), confidence, and safety concerns are some of the primary reasons for patients to be afraid or reluctant to mobilize in the ICU [34]. We hoped to encourage patients to conquer ICU-AW as well as critical illness; the sense of achievement when performing the activity might act as positive feedback for them to be more active in the mobilization program. These hypotheses are worthy of further validation by more studies.

EMN and EM had similar positive effect on ICU-AW and functional independence, and EMN had no significant advantages over EM and only led to a slightly lower chance of developing ICU-AW (approximately 93.5% less than that of standard care) than EM alone (approximately 93.4% less than that of standard care). However, participants in the EMN group were the only ones with improved muscle strength. One possible reason that EMN could enhance muscle strength improvement more than EM alone, but had no better results with respect to ICU-AW, is that ICU-AW was primarily diagnosed by the MRC sum score (< 48), which was the same tool used to assess muscle strength. Once the score reached 48 or over at ICU discharge, the fact that the patient did not have ICU-AW at the endpoint would not change regardless of how great the score reflecting his or her muscle strength was. A person with a score of 60 would have the same result with regard to the occurrence of ICU-AW as that of the one scoring 49. Nevertheless, the influence of early nutrition on the ICU-AW and functional status remains controversial across studies. In a multi-center RCT [35], early PN was found to be associated with a higher incidence of ICU-AW. Early PN may inhibit autophagic quality control of myofibers and increase intramuscular water/lipid content and the volume of adipose tissue islets, thus leading to weakness [36]. In another large-scale RCT, early PN reduced muscle wasting but did not affect functional status [37]. As for early EN, or early EN with PN if necessary, one study found that the incidence of ICU-AW was less [12], whereas another suggested that functional status was not affected by early EN [38]. In this trial, we conducted early nutrition as a part of EMN intervention following the ESPEN guideline [16], which suggests that nutritional support including oral feeding, EN, and PN is decided according to the patient’s condition, with oral feeding and EN given the top priority. Therefore, it is possible that early nutrition in this study might have little or even a negative influence on ICU-AW prevention and functional independence, and the result of EMN in this study may be mainly due to the application of EM. Furthermore, despite the existence of nutrition guidelines for the critically ill, observance of these guidelines remains poor [39]. In a cross-sectional study across China [40], EN was found to be suboptimal in ICUs and energy targets were poorly met during the early days of ICU stay. It is likely that early nutrition is still not prevalent in the ICU setting, and it needs to be implemented as an intervention to investigate its effect on physical functioning (including muscle strength, function, and muscle mass) [41] when applied alone compared to when implemented in EMN. More studies are required to further explore the impact of early nutrition alone compared to EMN.

Only the patients in the EMN group showing significant improvement in muscle strength following ICU admission after the trial could possibly be the result of mutual promotion between early nutrition and early mobilization. In this trial, EMN was found more likely to benefit patients’ nutritional status in comparison to routine care, whereas there was no statistical difference between EM and routine care. Early nutrition promotes nutritional status, preserves muscle mass, and provides the energy needed for exercise in the ICU, thus creating nutritional and muscular conditions suitable for mobilization and motivating the patients to participate in activities [12, 17]. Meanwhile, enhanced physical performance could help raise confidence in patients and reveal that they are actually getting better, which might stimulate them to face the critical illness more actively and increase their food intake to obtain an adequate amount of energy and protein for better performance [42]. As a result, it is possible that a positive feedback loop is established, leading to increased muscle strength and functional independence. Additionally, a systematic review suggested that enhanced nutritional intervention combined with structured mobilization intervention might increase muscle mass and functional independence [14]. However, only two studies were included in this review due to the lack of research concerning EMN intervention for ICU patients, and both studies only compared EMN with standard care, with the main focus of nutritional intervention being the effect of nutritional supplements (energy or amino acid supplements). Therefore, it is necessary to conduct further studies to compare the effects of EMN and EM.

Regarding the barriers to implementing EM in the ICUs, first, some patients initially had concerns about the safety of EM, such as displacement of their catheters, especially when they were undergoing continuous renal replacement therapy. Second, owing to safety concerns, some nurses were worried about the occurrence of adverse events. However, after a detailed explanation by the study team and seeing the successful and safe implementation of this intervention in other patients, the concerned nurses and patients gradually accepted the implementation of EM and took active part in the program. One major facilitator of the implementation of the EM/EMN protocol was that the departmental directors of the units attached great importance to EM and EMN and to this program; therefore, almost all ICU physicians and nurses participated actively in the EMAS trial, although their daily work was already burdensome.

Moreover, since this trial was conducted at two sites with a total of five ICUs, including both general and specialized ICUs, and the inclusion and exclusion criteria were defined clearly, ensuring the external validity of this study. Nevertheless, further validation of this trial is still required in other settings, such as other regions and other ICU settings (e.g., cardiac intensive care unit).

### Limitation

The EMAS trial had some limitations. First, we did not design an additional early nutrition intervention group because of the insufficient number of patients due to the COVID-19 pandemic. Second, some baseline variables related to ICU-AW, such as vasopressor support rates, the use of analgesics and sedatives, and the amount of exercise performed were not recorded. Daily caloric and protein intakes were not recorded, since all three groups followed routine standards for these aspects. Participants had a short ICU length of stay, which led to less time of exposure to the intervention, but the effects were still seen. Moreover, the assessors for the functional outcomes (MRC sum score and BI) could not be blinded due to limited resources and feasibility; however, each evaluation was completed by two therapists individually to control bias. Furthermore, follow-up was not conducted after ICU discharge, considering that there would be uncontrollable confounding factors outside the ICU [13]. Future studies should consider conducting follow-ups after ICU discharge, including health-related quality of life measures.

## Conclusions

In this study, early mobilization combined with early nutrition showed the potential to promote improvement in muscle strength and benefit nutritional status more than the usual care. In addition, compared with usual care, both EM and EMN might lead to a lower occurrence rate of ICU-AW and promote functional independence. Little difference was observed between the effects of EM and EMN. Further research is required to compare the effects of early nutrition with that of EMN and explore the long-term effects of EMN on critically ill patients.

## Supporting information

S1 Checklist EQUATOR checklist reporting of multi-arm parallel-group randomized trials: Extension of the CONSORT 2010 statement. (PDF)

S1 File Early mobilization intervention procedure and other information. (PDF)

S2 File Published protocol. (PDF)

S3 File Original study protocol submitted to the ethics review board (Chinese version). (PDF)

S4 File Original study protocol submitted to the ethics review board (English version). (PDF)

S5 File Clinical triage documentation. (PDF)

S1 Data (PDF)
