# Supplementary material for: Effect of early mobilization combined with early nutrition on acquired weakness in critically ill patients (EMAS): A dual-center, randomized controlled trial
Source: PLoS One. 2022 May 26;17(5):e0268599. doi: 10.1371/journal.pone.0268599 (PMC9135241; doi:10.1371/journal.pone.0268599)
Supplement: S1 Checklist — (PDF) [file pone.0268599.s001.pdf]

Table. Checklist for Reporting of Multi-Arm Parallel-Group Randomized Trials: Extension of the CONSORT 2010 Statement[a]

Section/Topic	Item No.	CONSORT 2010 Statement Checklist Item	Multi-Arm Trial Extension
Title and abstract			
	1a Title page	Identification as a randomized trial in the title	Identification as a multi-arm randomized trial in the title or an indication of the number of treatment groups that the participants were randomly assigned to
	1b Abstract	Structured summary of trial design, methods, results, and conclusions (for specific guidance see CONSORT for abstracts)[7]	Specification of the number of treatment groups; details of any groups added or dropped
Introduction			
Background and objectives	2a P5-6	Scientific background and explanation of rationale	Rationale for using a multi-arm design
	2b P6	Specific objectives or hypotheses	Specification of the research question referring to all of the treatment groups<br>Clear statement of all hypotheses to be tested and the primary comparisons involved
Methods			
Trial design	3a P6	Description of trial design (such as parallel, factorial) including allocation ratio	Specification of the number of treatment groups
	3b P6,10	Important changes to methods after trial commencement (such as eligibility criteria), with reasons	Details of any treatment groups added or dropped (if relevant), with reasons, and/or changes to the allocation ratio
Participants	4a P7-8	Eligibility criteria for participants	
	4b P6	Settings and locations where the data were collected	
Interventions	5 P8-11, S1 File	The interventions for each group with sufficient details to allow replication, including how and when they were actually administered	
Outcomes	6a P11-12	Completely defined prespecified primary and secondary outcome measures, including how and when they were assessed	
	6b NA	Any changes to trial outcomes after the trial commenced, with reasons	
Sample size	7a P12-13	How sample size was determined	Planned sample size with details of how it was determined for each primary comparison
	7b P13-14	When applicable, explanation of any interim analyses and stopping guidelines	
Randomization			
Sequence generation	8a P7	Method used to generate the random allocation sequence	
	8b P7	Type of randomization; details of any restriction, such as blocking and block size	
Allocation concealment mechanism	9 P7	Mechanism used to implement the random allocation sequence, such as sequentially numbered containers, describing any steps taken to conceal the sequence until interventions were assigned	
Implementation	10 P7	Who generated the random allocation sequence, who enrolled participants, and who assigned participants to interventions	

Blinding	11a P7	If applicable, who was blinded after assignment to interventions (eg, participants, care providers, individuals assessing outcomes) and how	
	11b P8-9	If relevant, description of the similarity of interventions	
Statistical methods	12a P13-14	Statistical methods used to compare groups for primary and secondary outcomes	Explicitly state if no adjustments for multiplicity were applied; if adjustments were applied, state the method used
	12b NA	Methods for additional analyses, such as subgroup analyses and adjusted analyses	
Results			
Participant flow (a diagram is strongly recommended)	13a Fig.1	For each group, the numbers of participants who were randomly assigned, received intended treatment, and were analyzed for the primary outcome	
	13b Fig.1	For each group, losses and exclusions after randomization with reasons included	
Recruitment	14a P14	Dates defining the periods of recruitment and follow-up	If periods of recruitment and follow-up are different across treatment groups (eg, groups were added or dropped), the periods of recruitment and follow-up, reason(s) for the differences, and any statistical implications should be described
	14b NA	Why the trial ended or was stopped	
Baseline data	15 Table 1	A table showing baseline demographic and clinical characteristics for each group	
Numbers analyzed	16 Fig.1	For each group, number of participants (denominator) included in each analysis and whether the analysis was by original assigned groups	
Outcomes and estimation	17a Table 2,3	For each primary and secondary outcome, results for each group, and the estimated effect size and its precision, such as 95% CI	Results for each prespecified comparison of treatment groups
	17b Table 2,3	For binary outcomes, presentation of both absolute and relative effect sizes is recommended	
Ancillary analyses	18 NA	Results of any other analyses performed, including subgroup analyses and adjusted analyses, distinguishing prespecified from exploratory	
Harms	19 NA	All important harms or unintended effects in each group (for specific guidance, see CONSORT for harms)[8]	
Discussion			
Limitations	20 P24-25	Trial limitations, addressing sources of potential bias, imprecision, and, if relevant, multiplicity of analyses	
Generalizability	21 P24	Generalizability (external validity, applicability) of the trial findings	
Interpretation	22 P19-24	Interpretation consistent with results, balancing benefits and harms, and considering other relevant evidence	
Other information			
Registration	23 P6	Registration number and name of trial registry	
Protocol	24 P6	Where the full trial protocol can be accessed, if available	
Funding	25	Sources of funding and other support (such as supply of drugs), role of funders Funding Statement section of the online submission form	

[a] It is strongly recommended that this checklist is read in conjunction with the CONSORT 2010 Statement Explanation and Elaboration[5] for important clarification on the items.
