# Supplementary material for: Effect of early mobilization combined with early nutrition on acquired weakness in critically ill patients (EMAS): A dual-center, randomized controlled trial
Source: PLoS One. 2022 May 26;17(5):e0268599. doi: 10.1371/journal.pone.0268599 (PMC9135241; doi:10.1371/journal.pone.0268599)
Supplement: S1 File — (PDF) [file pone.0268599.s002.pdf]

Early mobilization intervention modes

Mode 1: Wholly compensatory system

BI: < 40.

Exercises: Passive movement. Perform muscle kneading and passive movement of extremities twice per day. Exercises in the main direction of the joints of each limb were repeated 10 times, such as flexion and extension of upper limbs and fingers; flexion, extension, radial deviation and ulnar deviation of the wrist joint; flexion, extension, abduction and adduction of the elbow joint; and flexion, abduction, internal rotation and external rotation of the shoulder joint.

Mode 2: Partly compensatory system

BI: ≥40 and < 60.

Exercises: Passive movement combined with active movement. The passive exercise described in mode 1 was repeated 10-15 times with each limb. Clenching fists for 10 s and the ankle pump exercise for 15 s were both performed 15-40 times per side. Actively sit in bed for 5-15 min. If the patient was able to finish the above exercises, he or she would perform active movement of the joints of the extremities on the bed, that is, chest expansion, abduction of the upper limbs (arms) and kicking of the lower limbs (legs), all were repeated 15-35 times. Then the patient performed assisted bedside sitting for 5-20 min.

Mode 3: Supportive-educative system

BI: ≥60.

Exercises: Active movement. Deliver health education about active exercise and guide patients to learn to exercise independently. In addition to the active motion part in mode 2, the patient performed active bedside sitting for 5-10 min, and active standing against the bed for 5-10 min. Patients who could achieve these would shift to taking a few steps in one place with assistance

for 15-25 times, then to standing independently for 5 min. Those who could accomplish the above exercises, would walk for 5-20 min under the guidance of the physiotherapist.

The mobilization session would be paused or terminated if the patient [1, 2]:

- Had a heart rate above 130 bpm/min or below 60 bpm/min
- Had a heart rate decreasing by more than 20% while resting, with irregular rhythm
- Had a systolic blood pressure above 180 mmHg or below 90 bpm/min, or mean arterial pressure above 100 mmHg or below 60 mmHg
- Had a blood oxygen saturation below 88%
- Had a respiratory rate below 5 breaths/min or above 40 breaths/min
- Received MV, and the oxygen concentration was above 60%; or the positive end expiratory pressure (PEEP) was above 10 cmH_2O, and the patient was ventilated by control mode (CMV)
- Had disorders of consciousness, such as disobeying instructions, euphoria, pugnacity, or irritability; new-onset arrhythmia requiring vasoactive drug maintenance, chest pain with myocardial ischemia, man-machine confrontation, falling, bleeding, medical device removal or failure, respiratory distress (self-reported and/or observed by medical staff)

Table S1. Personnel and tasks of the interventions.

Interventions	Timepoint	Personnel	Task
Early mobilization (EM)	Before the intervention	Physiotherapist, ICU doctor, responsible nurse and researcher	Evaluation of the vital signs, condition, BI and MRC sum score of the patient[a]
	During the intervention	Physiotherapist	Taking charge of the mobilization, abiding by the paused/terminated criteria
		Researcher	Recording the form and situation of the patient's activities
	After the intervention	Responsible nurse and researcher	Evaluation of the safety of the mobilization
	Throughout the intervention	Head nurse	Supervision of the implementation of the EM, quality control
Early nutrition	Before the intervention	ICU doctor, dietician and researcher	Evaluation of the nutritional status and condition of the patient, determining the start time and route of nutritional support
	During the intervention	Responsible nurse	Operation and monitoring of nutritional support
	After the intervention		
	Throughout the intervention	Head nurse	Supervision of the implementation of the early nutrition, quality control

BI, Barthel Index; ICU, intensive care unit; MRC, Medical Research Council

[a] Two physiotherapists assessed the MRC sum score and BI individually

Table S2. Outcome assessment.

Assessment	Instrument	Timepoint
**Primary outcome**		
Occurrence of ICU-AW	MRC sum score	ICU discharge
**Secondary outcomes**		
Overall muscle strength	MRC sum score	Enrolment, every 24 h following enrollment, ICU discharge
Functional independence	Barthel Index	Enrolment, every 24 h following enrollment, ICU discharge
Organ failure	SOFA	Enrolment, every 24 h following enrollment, ICU discharge
Nutritional status	SGA	Enrolment, ICU discharge
Duration of MV	Chart review	ICU discharge
Length of ICU stay	Chart review	ICU discharge
ICU mortality	Chart review	ICU discharge

ICU, intensive care unit; ICU-AW, intensive care unit-acquired weakness; MRC, Medical Research Council; MV, mechanical ventilation; SGA, subjective global assessment; SOFA, sequential organ failure assessment

REFERENCES

1. Hodgson CL, Stiller K, Needham DM, Tipping CJ, Harrold M, Baldwin CE, et al. Expert consensus and recommendations on safety criteria for active mobilization of mechanically ventilated critically ill adults. Critical care (London, England). 2014;18(6):658.

2. Han R, Lee X, Zhao S, Lee Y, Chen L, Duan H, et al. Construction and application of early rehabilitation scheme for Intensive Care Unit patients. Chin J Nurs. 2020;55(1):8-15.
