# Supplementary material for: Effect of early mobilization combined with early nutrition on acquired weakness in critically ill patients (EMAS): A dual-center, randomized controlled trial
Source: PLoS One. 2022 May 26;17(5):e0268599. doi: 10.1371/journal.pone.0268599 (PMC9135241; doi:10.1371/journal.pone.0268599)
Supplement: S2 File — (PDF) [file pone.0268599.s003.pdf]

Study Protocol Clinical Trial

Medicine®

OPEN

Effect of early activity combined with early nutrition on acquired weakness in ICU patients

Wendie Zhou, BS[a,b], Baisheng Shi, MD[c], Yuying Fan, MD, PhD[a,b,*], Jinsong Zhu, MD[d]

Abstract

Introduction: Intensive care unit-acquired weakness (ICU-AW) occurs in 25% to 100% of critically ill patients, and is associated with prolonged mechanical ventilation, extended ICU stay, and total hospital stay, increased hospital costs, higher risk of death, impaired physical function, and decreased quality of life. However, there are not any current guidelines that mention management of ICU-AW. The present study will evaluate the effects of a combination of early nutrition and early exercise compared to those of either early exercise alone or the standard care for patients in ICUs.

Methods: This is a 3-arm, parallel, randomized controlled trial including an estimated 147 critically ill patients aged ≥18 years recruited from the ICUs of 2 hospitals in Heilongjiang, China. Patients will be prospectively randomized 1:1:1 to receive early mobilization, early nutrition combined with early mobilization, or standard care (minimal exercises, experience-based initiation and enrollment of nutrition support). Outcomes are assessed at ICU discharge after baseline. The primary outcome is occurrence of ICU-AW according to the Medical Research Council scale at the end of treatment. Muscle strength, organ failure, functional independence, self-care ability, time of ICU stay, duration of mechanical ventilation, and ICU mortality are secondary outcome measures.

Discussion: This trial has the potential to identify a novel strategy for preventing or managing ICU-AW. The findings may increase the clinical knowledge about nutrition and mobilization interventions for people with ICU-AW, and contribute to the formation of practice guidelines for managing this condition.

Trial registration number: ChiCTR2000033482

Abbreviations: BI = Barthel index, CVP = central venous pressure, EN = enteral nutrition, ICU = intensive care unit, ICU-AW = intensive care unit-acquired weakness, MRC = Medical Research Council, PN = parenteral nutrition, RCT = randomized controlled trial, SGA = subjective global assessment, SOFA = sequential organ failure assessment.

Keywords: critical illness, early mobilization, intensive care unit-acquired weakness, muscle weakness, nutrition, randomized controlled trial (topic)

Downloaded from http://journals.lww.com/md-journal by B+VcUVcwNJqQUSfjKtzR0cSrJ0GlM5bqqX6fpxU7TMeldJV38gcSBA/3Q2s0l2qxEs1ZesrLuR2k+bxlv+D9GMGjuLUTRB0ljPDYzCbsEYyV9P41+Mmdw4G7etzjM3IZHYWO8A= on 07/17/2020

1. Introduction

Intensive care unit-acquired weakness (ICU-AW) refers to the loss of muscle function and acute nerve damage or loss in ICU patients without a clear cause, including critical myopathy, multiple neurosis, or a combination of both.[1] Risk factors for ICU-AW include sepsis, trauma, injury, tumor, renal and liver failure, immobilization (due to bed rest, deep sedation, and neuromuscular blockade), malnutrition, severity of disease, aminoglycosides, female sex, multiple organ failure, systemic inflammatory response syndrome, electrolyte disturbances, and hyperglycemia.[2,3] Several studies have shown that ICU-AW occurs in 25% to 100% of critically ill patients.[4] Although the establishment of ICUs has significantly reduced mortality from nosocomial infections and severe diseases, the number of people transferred directly from the ICU to rehabilitation centers has tripled during this period.[5] Moreover, evidence indicates that 60% to 80% of ICU "survivors" will suffer functional impairment or ICU-AW.[5] ICU-AW is associated with prolonged mechanical ventilation, extended ICU stay and total hospital stay, increased hospital costs associated with health care, higher risk of death, impaired body function, and decreased quality of life in the months following ICU admission.[6] Patients with ICU-AW often sustain persistent disability after discharge from the ICU, and of these, one third or more will need permanent personal assistance 1 year after discharge; only about 50% are able to recover completely.[7]

It is increasingly recognized, and supported by systematic reviews,[8–10] that early mobilization plays a significant role in preventing ICU-AW. In a setting where early rehabilitation is already utilized in clinical practice, the addition of even 1 day of mobilization therapy decreases length of stay, improves func-

The authors have no conflicts of interest to disclose

The datasets generated during and/or analyzed during the current study are available from the corresponding author on reasonable request.

[a] The Second Affiliated Hospital of Harbin Medical University, [b] School of Nursing, Harbin Medical University, [c] Department of Rehabilitation, the Second Affiliated Hospital of Harbin Medical University, Harbin city, [d] Nursing Department, the First Affiliated Hospital of Jiamusi University, Jiamusi city, China.

[*] Correspondence: Yuying Fan, The Second Affiliated Hospital of Harbin Medical University & School of Nursing, Harbin Medical University, No. 194, Bao Jian Road, Nan-Gang District, Harbin 150086, China (e-mail: fanfanfensituan@126.com).

Copyright © 2020 the Author(s). Published by Wolters Kluwer Health, Inc. This is an open access article distributed under the Creative Commons Attribution License 4.0 (CCBY), which permits unrestricted use, distribution, and reproduction in any medium, provided the original work is properly cited.

How to cite this article: Zhou W, Shi B, Fan Y, Zhu J. Effect of early activity combined with early nutrition on acquired weakness in ICU patients. Medicine 2020;99:29(e21282).

Received: 12 June 2020 / Accepted: 15 June 2020

http://dx.doi.org/10.1097/MD.0000000000021282

tional independence, and provides benefits that last to the 6-month follow-up.[11] Similar to the role of early mobilization, nearly all current guidelines for nutrition in critically ill patients recommend early initiation of nutrition.[12,13] Several studies have explored the influence of early nutrition on ICU-AW.[14–16] A randomized controlled trial (RCT) showed a relationship between early enteral nutrition (EN) and reduced weakness.[14] Early nutrition can suppress the extent of tissue damage caused by the inflammatory response by inhibiting excessive immune responses.[12] However, a multicenter RCT found that the implementation of early parenteral nutrition (PN) was related to increased weakness.[15] This could be due to the fact that macronutrients at the early stage of critical illness may suppress muscle autophagy.[16] However, this hypothesis of restrained autophagy needs to be evaluated in future trials.[17,18]

Early EN is beneficial and should not be restricted.[19] Moreover, a combination of nutrition therapy with exercise may have the greatest potential to prevent or attenuate ICU-AW and benefit the physical recovery of survivors of critical illness, as suggested not only by previous studies[6,20,21] but also by clinical guidelines.[13] The vast majority of patients admitted to ICUs are at risk for malnutrition, which may contribute to muscle weakness.[20] Moreover, nutritional support for critically ill patients in the ICU can enhance their enthusiasm to participate in exercise.[20] Nutritional treatment combined with early activity can limit the loss of muscle mass, inhibit the development of ICU-AW, reduce the associated impairment of body function, decrease patient mortality, and accelerate recovery from disease.[6,20] Nevertheless, the application of this innovative methodology is still rare. To date, a limited number of studies have investigated the effectiveness of a joint intervention including both early activity and nutrition in critically ill patients for the purpose of improving patient rehabilitation in terms of ICU-AW.

The present study will be a multicentered, randomized controlled trial with 3 parallel groups. The experimental group will receive either early nutrition combined with early mobilization or early mobilization alone. The control group will receive standard care only. Considering that the promising effects of early mobilization on ICU-AW are widely acknowledged, this study aims to investigate whether the addition of early nutrition would be more effective than the former alone. This novel combined intervention design is still in the conceptual phase, not yet being validated by sufficient RCTs. This study hypothesizes that a combined nutrition and mobilization intervention, compared with early mobilization alone or standard care, will reduce the development of ICU-AW and improve the daily self-care ability of patients at ICU discharge. The findings of this research may provide evidence for future guidelines on the prevention of acquired weakness in ICU patients.

2. Materials and methods

2.1. Study design

This is a multicenter, single-blinded, RCT conducted at the ICUs of the Second Affiliated Hospital of Harbin Medical University and the First Affiliated Hospital of Jiamusi University in China. Participants will be randomized to 1 of 3 conditions: standard ICU care, early mobilization added to standard care (EM group), or early nutrition combined with early mobilization in addition to the standard care (ENM group). Figure 1 shows the study flow diagram. The research protocol is registered at chictr.org.cn (ChiCTR2000033482). Ethical approval was granted by the ethics committee of the Second Affiliated Hospital of Harbin Medical University (KY 2020–012).

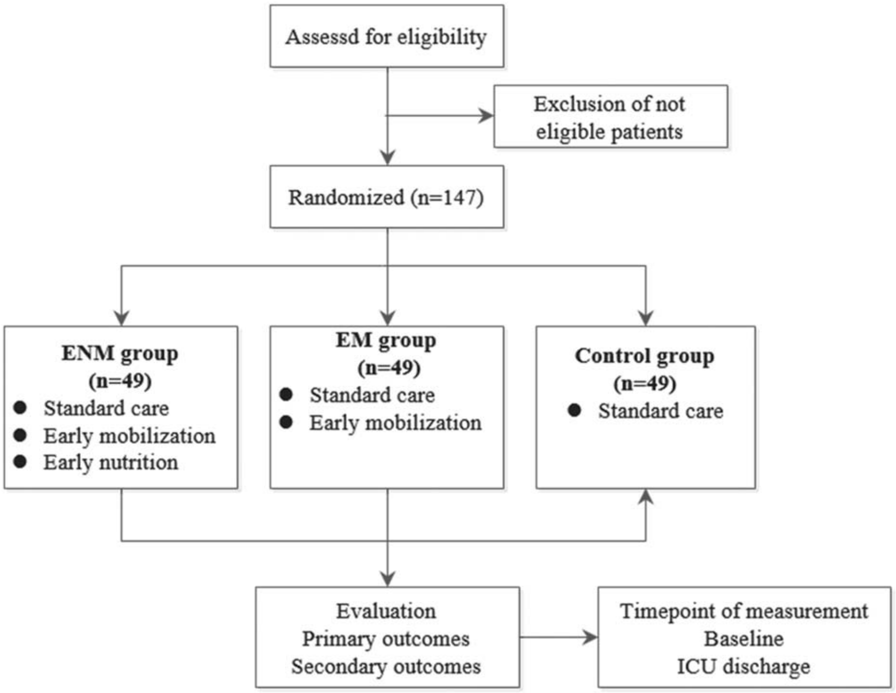

Figure 1. Study flow diagram. EM = early mobilization, ENM = early nutrition and early mobilization, ICU = intensive care unit.

Table 1

Inclusion and exclusion criteria.

Inclusion criteria	Exclusion criteria
Age ≥18 yr	Incomplete limbs
Admitted to ICU for the first time	Pre-existing primary systemic neuromuscular disease (e.g., Guillain-Barre, myasthenia gravis, amyotrophic lateral sclerosis)
Expected ICU stay ≥72 h	Intracranial or spinal process affecting motor function
Consciousness adequate to respond to at least 3 of these orders: "open/close your eyes," "look at me," "put out your tongue." "nod your head," "raise your eyebrows"	Had gastrointestinal surgery in 1 mo
Informed consent of patients or their agents	Hyperthyroidism and other endocrine diseases affecting nutrition and metabolism
	No expectation for any nutritional intake within the subsequent 48 h
	Pregnant
	Has terminal cancer
	Expected death or extremely poor prognosis

APACHE = acute physiology and chronic health evaluation, ICU = intensive care unit, NRS = Nutrition Risk Screening.

2.2. Participants and settings

The study will enroll at least 147 men and women who are aged 18 or older and admitted to the ICU. We expect our intervention to be most effective when delivered early; thus, we will limit enrolment to the first 24 hours after ICU admission. Eligible participants must be conscious within the first 24 hours of ICU stay, as muscle strength and ICU-AW are assessed by the Medical Research Council (MRC) sum score, which requires patients to be awake and attentive during the examination;[22] the level of consciousness must be adequate for responding to at least 3 of the following orders: "open/close your eyes," "look at me," "put out your tongue," "nod your head," "raise your eyebrows."[23] ICU-AW is diagnosed when a patient has an MRC sum score of less than 48.[24] Patients expected to stay in the ICU for less than 72 hours after enrolment will be excluded to allow for adequate exposure to the proposed intervention. Inclusion and exclusion criteria are summarized in Table 1.

There are no nutrition or physiotherapy protocols in the units, and the units do not have a physiotherapist available at all times. When the doctor believes a patient would benefit from physiotherapy based on his or her experience, he or she will notify the patient's family, who will decide whether to proceed with the therapy for the patient. If the family's answer is affirmative, the physiotherapist from the rehabilitation department will be invited to begin rehabilitation treatment with the patient. The rehabilitation methods vary by therapist. For instance, some perform only passive movement on the bed for all patients, while others adjust their strategy based on the patient's present condition. Overall, however, exercise for patients in the units is very minimal. The initiation of and methods for nutrition therapy (i.e., time and nutrition route) will be based on the doctors' experience. Enteral nutritional suspension or enteral nutritional suspension is used for enteral feeding. KABIVEN (amino acids, electrolytes, dextrose, and lipid injectable emulsion) is applied for parenteral nutrition.

2.3. Intervention

All 3 groups will receive standard ICU care, which includes conventional nutrition support and physiotherapy. The conventional physiotherapy, performed once per day without a regular time or frequency (i.e., in the morning or in the afternoon, every day, every other day, or once a week), is not applied to all patients, but rather for only a few specific patients based on the doctors' judgment. When the patient is in the EM or ENM group and is simultaneously receiving conventional physiotherapy, the early mobilization intervention will be performed once on that day, and thus the patient will receive physiotherapy twice per day. If the conventional therapy is performed in the morning, the intervention will be carried out in the afternoon, and vice versa. Patients in the intervention groups who are not receiving conventional physiotherapy will receive the early exercise therapy twice daily, both in the morning and afternoon.

With the exception of patients in the ENM group, all patients in the other 2 groups will receive conventional nutrition therapy. The nutrition therapy components (i.e., brand, composition) and feeding rate will be the same in all 3 groups. The energy consumption equation will be identical. The management of oral nutritional, EN, and PN in the 3 groups will be the same except for the time of initiation. Patients who require EN will all receive continuous feeding.

2.3.1. Early mobilization.

In the interest of individualized mobilization, the early mobilization intervention is based on the theory of nursing systems of Orem's theoretical framework, which divides modes of nursing care into 3 categories: the wholly compensatory system, the partly compensatory system, and the supportive-educative system.[25] The mobilization mode that the patient receives is decided by his or her independent functional status, which is measured by the Barthel index (BI).[26] The BI is an ordinal scale consisting of 10 activities of daily living. The items can be categorized into those related to self-care (feeding, grooming, bathing, dressing, bowel and bladder care, and toilet use) and those related to mobility (ambulation, transfers, and stair climbing). Scores range from 0 (disabled) to 100 (very independent).[27] A score of more than 60 indicates that there is a mild dysfunction, which means the patient can independently complete some of the daily activities and a certain amount of help is needed; a score of 59 to 41 indicates that there is a moderate dysfunction, which suggests the patient requires considerable help to complete daily activities; and a score of less than 40 indicates that there is a severe dysfunction, suggesting that most of the daily activities cannot be completed by the patient.[25]

Patients in the ENM and EM groups will receive early mobilization intervention twice daily in addition to standard treatment. The early mobilization intervention is created by the

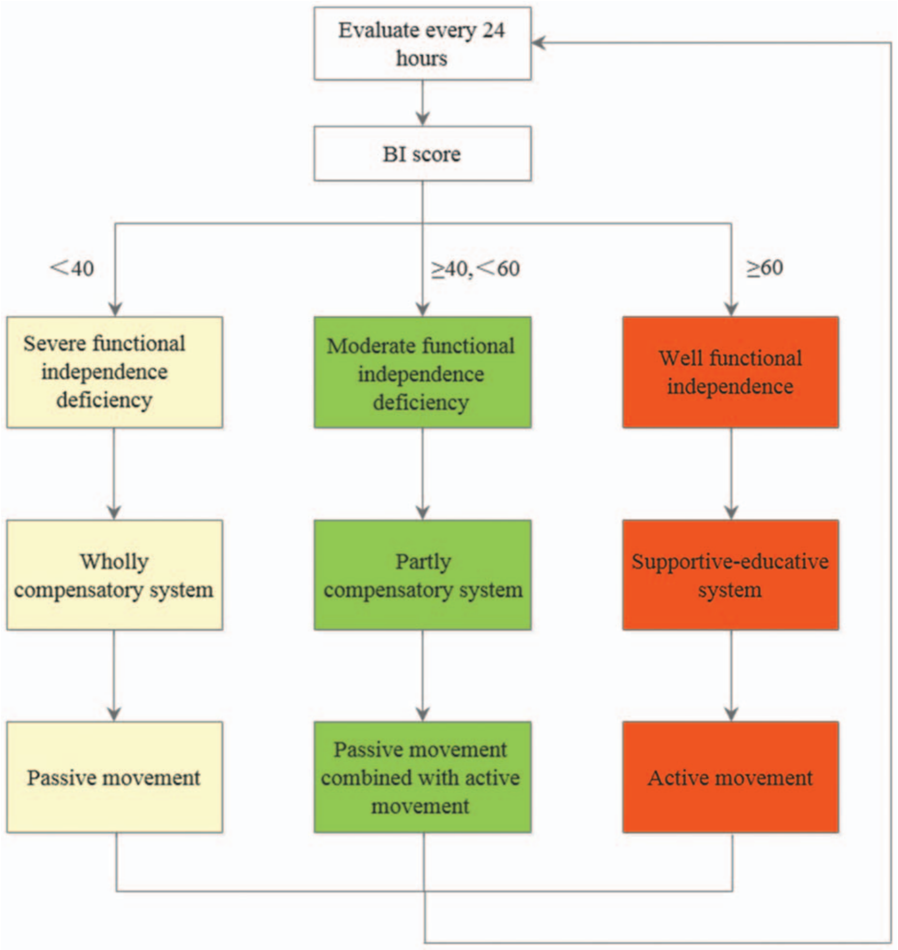

Figure 2. The early mobilization intervention. BI=Barthel index.

research team based on data in the academic literature. Physiotherapists, nurses responsible for the patients, and the study researchers will collaborate to implement early exercise intervention strategies based on the self-care theory, starting within 24 hours of ICU admission. The physiotherapist and researcher will evaluate the patient's functional status, choose the appropriate exercise strategy accordingly, and perform the intervention with the nurse who is in charge of the patient. The BI score evaluation will be conducted every 24 hours, and will commence with the first intervention. The 3 modes of the early mobilization program are shown in Figure 2. Based on the patient's BI score, the assessor will determine whether or not to change the mobilization mode.

2.3.2. Mode 1: Wholly compensatory system

2.3.2.1. BI score: <40. Exercises: Passive movement. Perform muscle kneading and passive movement of extremities twice per day. Exercise in the main direction of the joints of each limb will be repeated 10 times, such as flexion and extension of upper limbs and fingers; flexion, extension, radial deviation, and ulnar deviation of the wrist joint; flexion, extension, abduction, and adduction of the elbow joint; and flexion, abduction, internal rotation, and external rotation of the shoulder joint.

2.3.3. Mode 2: Partly compensatory system

2.3.3.1. BI score: ≥40 and <60. Exercises: Passive movement combined with active movement. The bed-head angle will be increased to 30° to 45°, and the passive exercise described in mode one will be repeated 5 times with each limb. Clenching fists for 10 seconds and the ankle pump exercise for 15 seconds will both be performed 20 times per side. Active sitting in bed will be performed for 20 minutes. If the patient is able to finish the above exercises, he or she will perform active movement of the joints of the extremities while on the bed; that is, chest expansion and abduction of the upper limbs (arms) and kicking of the lower limbs (legs), all repeated 30 times. Patient will then perform assisted bedside sitting for 20 minutes. Patients capable of these movements will progress to assisted standing against the bed for 5 minutes.

2.3.4. Mode 3: Supportive-educative system

2.3.4.1. BI score: ≥60. Exercises: Active movement. Patient will perform active bedside sitting for 10 minutes, and active standing against the bed for 10 minutes. Patients who can achieve these will shift to standing independently for 5 minutes, then marking time for 10 minutes.

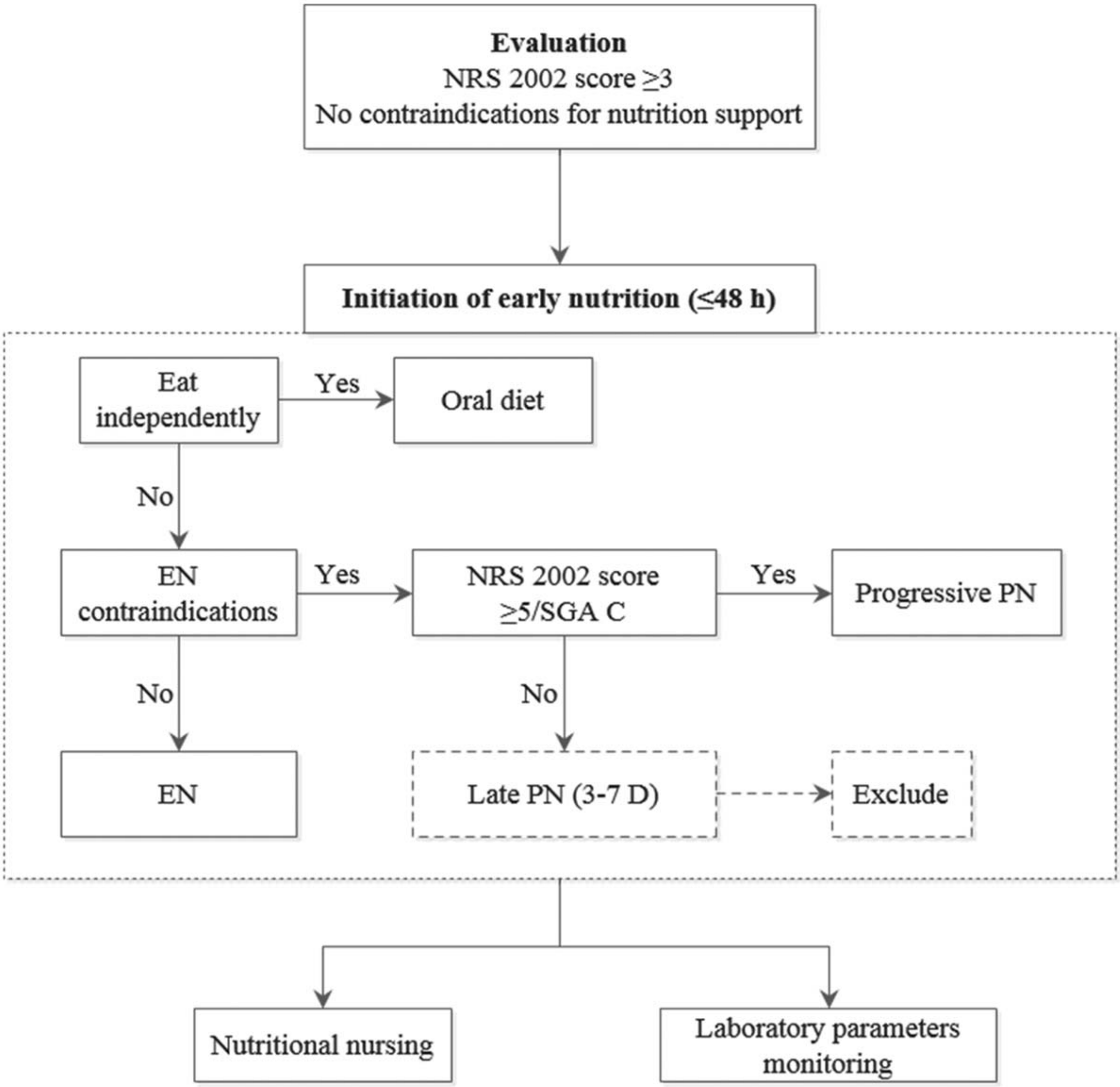

Figure 3. The early nutrition intervention. EN = enteral nutrition, NRS = Nutrition Risk Screening, PN = parenteral nutrition, SGA C = grade C of the subjective global assessment.

The therapy session will be paused or terminated if the patient:

- Has a heart rate above 130 bpm/min or below 60 bpm/min
- Has a heart rate decreasing by more than 20% while resting, with irregular rhythm
- Has a systolic blood pressure above 180 mm Hg or below 90 mm Hg, or mean arterial pressure above 100 mm Hg or below 60 mm Hg
- Has a blood oxygen saturation below 88%
- Has a respiratory rate below 5 breaths/min or above 40 breaths/min
- Receives mechanical ventilation, and the oxygen concentration is above 60%; or the positive end expiratory pressure is above 10 cm H_2O, and the patient is ventilated by control mode (CMV)

2.3.5. Early nutrition. In addition to ICU routine nursing and the early mobilization intervention, patients in the ENM group will receive an early medical nutrition program based on the 2018 European Society for Clinical Nutrition and Metabolism guideline[13] and the academic literature; this will be initiated within 48 hours of ICU admission (Fig. 3).

Patients with an Nutrition Risk Screening 2002 score ≥3, which indicates the need for nutrition support,[28] will receive early medical nutrition therapy consisting of early oral nutritional supplements and artificial nutrition (EN, parenteral nutrition).[13] Oral feeding is preferred over artificial nutrition when the patient is able to eat independently. Patients who cannot take food by mouth will be given early persistent EN within 48 hours. Postoperative patients will be supplied with EN within 24 hours after surgery. Early EN is preferred over early (PN). Patients with severe malnutrition (grade C of the subjective global assessment)[29] or with high nutrition risk (Nutrition Risk Screening 2002 score ≥5)[28] will receive early progressive low-dose PN if they have EN contraindications. At the early stage of the acute phase of injury (within 3 days), patients will be provided

Table 2

Primary and secondary outcomes.

Assessment	Instrument	Timepoint
Primary outcome		
Occurrence of ICU-AW	MRC sum-score	ICU discharge
Secondary outcomes		
Overall muscle strength	MRC sum-score	Enrolment, every 24 h following enrollment, ICU discharge
Organ failure	SOFA score	Enrolment, ICU discharge
Functional independence	BI	Enrolment, every 24 h following enrollment, ICU discharge
Self-care ability	BI	Enrolment, ICU discharge
Time of ICU stay	Chart review	ICU discharge
Duration of mechanical ventilation (survivors, non-survivors)	Chart review	ICU discharge
ICU mortality	Chart review	ICU discharge

BI = Barthel index, ICU = intensive care unit, ICU-AW = intensive care unit-acquired weakness, MRC = Medical Research Council, SOFA = sequential organ failure assessment.

with low-calorie nutrition of no more than 70% of their measured energy consumption. After 3 days, the amount of calories given should be increased to 80% of the measured energy consumption. Patients allocated to the ENM group will have energy requirements calculated as 20 to 25 kcal/kg/d.

When implementing EN during the early nutrition intervention, the responsible nurses will make sure that the gastric tube or other feeding tubes remain in proper position, and the gastric residual volume is less than 500 mL (to be checked every 6 hours). The head of the bed should be raised by 30° to 45°. The patient should be fed at the rate of 20 mL/h and monitored for gastrointestinal syndrome (diarrhea, abdominal distension, abdominal pain, nausea, or vomiting, etc). The feeding speed should be adjusted according to the patient's condition and reaction. Feeding should be stopped during exercise to prevent accidental inhalation.

Blood glucose level will be measured at ICU admission and at least every 4 hours thereafter during the first 2 days. The blood glucose level should be maintained between 6 and 10 mmol/L, with insulin applied if necessary.[30,31] Blood electrolytes (potassium, magnesium, phosphorus) will be monitored at least once per day during the first week. For patients with refeeding hypophosphatemia (serum phosphorus < 0.65 mmol/L or decreased by more than 0.16 mmol/L), electrolytes should be checked twice per day and phosphorus supplemented if necessary.

2.3.6. Control.

Participants in the control group will only receive standard ICU care. The usual care strategy is as follows:

1. Closely monitor vital signs, observe any changes in illness; regularly measure central venous pressure, arterial blood pressure, and blood gas analysis; identify and manage abnormal conditions in a timely manner.
2. Maintain indoor temperature and humidity at suitable levels and keep the bed clean and tidy.
3. Carefully manage all types of tubes.
4. Perform oral nursing care, perineal care, bedsore nursing; turn patient over every 2 hours.
5. Invite the occupational therapist, according to the ICU doctor's order, to perform rehabilitation exercise therapy for certain patients with no fixed time, method, or frequency, as described above. The present conventional methods include massaging muscles; passive, active assisted, or active mobilization; and bed positioning and maintenance of orthostatism. Nevertheless, the type of therapy will not be defined in advance, but instead will be at the discretion of the attending physiotherapist and will not have a pre-established routine.
6. Provide nutritional support as ordered by the doctors. Energy requirements are calculated as 20 to 25 kcal/kg/d. Different doctors have different strategies of nutrition support, such as initiation time, route of nutrition, and inclusion or exclusion criteria for nutrition therapy.
7. Relatives of patients can visit once daily, 10 minutes per visit.

2.4. Outcomes

In the development of our evaluation framework, we referred to the recommendations of a recent expert consensus statement.[6] The primary aim of this study is to measure in-patient muscle weakness, physical functioning, and functional independence. This will be evaluated by one primary outcome and multiple secondary outcomes. The timing of these evaluations is detailed in Table 2. Considering that the risk of muscle wasting, weakness, and physical impairment may be less after critical illness has been resolved,[6] as well as the high number of interfering factors beyond our control after ICU discharge, we decided to cease the intervention and outcome evaluation at the time of ICU discharge.

The primary outcome will be the incidence of ICU-AW, diagnosed with the MRC sum score for the clinical evaluation of muscle strength at ICU discharge. Muscle weakness using the MRC sum score is evaluated via standardized "manual muscle testing" with each of the 12 muscle groups assessed using a 6-point MRC scale and summed to a total score (range: 0–60).[32] The secondary outcomes include overall muscle strength measured by MRC sum score, organ failure by sequential organ failure assessment score, functional independence and self-care ability by BI score, length of ICU stay, duration of mechanical ventilation (survivors and non-survivors), and ICU mortality. sequential organ failure assessment score[33] is a validated composite score of 6 organ systems used to assess the severity of ICU organ dysfunction, and may correlate with loss of muscle mass.[34]

2.5. Statistical analysis including sample size calculations

Analyses for all outcomes will be performed according to the intention-to-treat paradigm. Comparative analysis between the

control group and the intervention group will be performed for all outcome variables. For the comparison of physiological variables and the levels of activity between groups, a statistical test will be used to compare the 2 independent groups. For parametric data, the *t* test will be used.

With a power of 90% and 2-sided 5% level of significance, 49 participants per group (147 total) will be needed to detect a significant between-group difference in the primary outcome, as calculated by PASS (version 11.0.7) and allowing for up to 10% attrition at ICU discharge (i.e., 132 participants with complete data). The estimated effect size is based on the incidence of ICU-AW at ICU discharge (early mobilization group=33.1%, control group=51.9%) reported in a meta-analysis of early mobilization interventions for critically ill patients[9] as well as the findings of Chen (2017) in a clinical trial (15.9%).[35]

2.6. Randomization and blinding

Recruitment sessions (n=147) will be randomized in a 1:1:1 ratio to the EM group, the ENM group, and the control group. The randomization sequence was generated by a non-investigator using Microsoft Office Excel. The specific operations are as follows: open Excel to input the number according to the determined sample size in the first column; enter "RAND ()" in the second column to get the random number 1; copy the random number 1 of the second column (copying only the numerical value) to the third column to get the random number 2. The random number 2 values were used in ascending order, the first 1/3 as the control group, the middle 1/3 as the EM group, and the latter 1/3 as the ENM group, and then arranged in ascending order with the number of the first column to form the final random grouping.

The group allocation will be concealed from the recruiters until the beginning of each recruitment session. Blinding of participants and study personnel is not possible for behavioral interventions; however, the participants will not be informed about the treatment received by other groups, and the curtains beside the bed will be closed during implementation of the early mobilization intervention to prevent contamination and ensure blinding of the other patients. Patients will not be given a definite answer about his or her allocation, as the care personnel are not allowed to leak any information. The statistical analyst will be blinded to the group allocation.

3. Discussion

This early nutrition combined with early mobilization in ICU-acquired weakness (EMAS) trial will be the first randomized trial of a combined early intervention design with 3 parallel groups in the context of ICU-AW. The primary objective of this study will be to compare the use of a combined early nutrition and early mobility program designed to prevent muscle weakness with an early mobility program only and a conventional therapy program, assessing the benefits of the combined intervention for the muscular and functional systems of patients in the ICU. To achieve individualized mobilization, we designed the EMAS trial based on the novel application of the theory of nursing systems in Orem's theoretical framework, which has not been previously utilized in a mobilization intervention for ICU patients; this design allows for patients to have exercises designed to suit their individual functional level. Additionally, the use of ascending levels of modes of mobilization guarantee, to some extent, progressive exercises. We hope that, by applying Orem's self-care theory, patients will gain awareness of moving independently, regain self-care abilities, and realize that they themselves play an indispensable role in overcoming critical illness.

The limitations of this study include the inability to blind the study interventions, due to the nature of behavioral interventions. Moreover, the assessor cannot be blinded due to limited resources and feasibility. Accordingly, the outcome analyst will be blinded to prevent the patient from inadvertently discovering the exact group he or others are allocated to. Another limitation of this protocol is that neither the intervention nor the follow-up will be continued after ICU discharge due to reduced functional impairment in the absence of critical illness and uncontrollable confounding variables outside of the ICU. Finally, the small sample size and the limited number of study sites limit the generalizability of our findings.

Despite these limitations, this trial has the potential to identify a novel strategy for preventing or managing ICU-AW. Our findings can inform clinical practice and future research in the management of ICU-AW regarding the effectiveness of combining early nutrition with early mobilization and the importance of motivating patients to participate in their own rehabilitation, thus, benefiting both patients and the health care system.

Acknowledgments

We would like to thank the support of the medical staff in the ICU of the Second Affiliated Hospital of Harbin Medical University and the First Affiliated Hospital of Jiamusi University. We thank Editage's (www.editage.cn) English language editing.

Author contributions

Wendie Zhou, Baisheng Shi and Jinsong Zhu contributed equally to this work and should be considered co-first authors. Wendie Zhou conceived the study, wrote the study protocol, and will conduct the intervention. Baisheng Shi contributed to the study design and will conduct the intervention. Yuying Fan contributed to the study design and reviewed the study protocol. Jinsong Zhu reviewed the study protocol and will conduct the intervention. All authors have approved the final version of manuscript.

References

[1] Intiso D, Amoruso L, Zarrelli M, et al. Long-term functional outcome and health status of patients with critical illness polyneuromyopathy. Acta Neurol Scand 2011;123:211–9.
[2] Yang T, Li Z, Jiang L, et al. Risk factors for intensive care unit-acquired weakness: a systematic review and meta-analysis. Acta Neurol Scand 2018;138:104–14.
[3] Latronico N, Bolton CF. Critical illness polyneuropathy and myopathy: a major cause of muscle weakness and paralysis. Lancet Neurol 2011; 10:931–41.
[4] Zorowitz RD. ICU-acquired weakness: a rehabilitation perspective of diagnosis, treatment, and functional management. Chest 2016;150: 966–71.
[5] Wischmeyer PE, San-Millan I. Winning the war against ICU-acquired weakness: new innovations in nutrition and exercise physiology. Crit Care (London, England) 2015;3(Suppl 19):S6.
[6] Heyland DK, Stapleton RD, Mourtzakis M, et al. Combining nutrition and exercise to optimize survival and recovery from critical illness: conceptual and methodological issues. Clin Nutr (Edinburgh, Scotland) 2016;35:1196–206.
[7] Veldema J, Bosl K, Kugler P, et al. Cycle ergometer training vs resistance training in ICU-acquired weakness. Acta Neurol Scand 2019;140: 62–71.

[8] Fuke R, Hifumi T, Kondo Y, et al. Early rehabilitation to prevent postintensive care syndrome in patients with critical illness: a systematic review and meta-analysis. BMJ Open 2018;8:e019998.
[9] Zang K, Chen B, Wang M, et al. The effect of early mobilization in critically ill patients: a meta-analysis. Nurs Crit Care 2019;1–8. https://doi.org/10.1111/nicc.12455.
[10] Zhang L, Hu W, Cai Z, et al. Early mobilization of critically ill patients in the intensive care unit: a systematic review and meta-analysis. PloS One 2019;14:e0223185.
[11] Peiris CL, Taylor NF, Shields N. Additional Saturday allied health services increase habitual physical activity among patients receiving inpatient rehabilitation for lower limb orthopedic conditions: a randomized controlled trial. Arch Phys Med Rehabil 2012;93:1365–70.
[12] Reintam Blaser A, Starkopf J, Alhazzani W, et al. E.W.G.O.G. Function, early enteral nutrition in critically ill patients: ESICM clinical practice guidelines. Intensive Care Med 2017;43:380–98.
[13] Singer P, Blaser AR, Berger MM, et al. ESPEN guideline on clinical nutrition in the intensive care unit. Clin Nutr (Edinburgh, Scotland) 2019;38:48–79.
[14] Liu Y, Zhao W, Chen W, et al. Effects of early enteral nutrition on immune function and prognosis of patients with sepsis on mechanical ventilation. J Intensive Care Med 2018;1–9. doi:10.1177/0885066618809893.
[15] Hermans G, Casaer MP, Clerckx B, et al. Effect of tolerating macronutrient deficit on the development of intensive-care unit acquired weakness: a subanalysis of the EPANIC trial. Lancet Respir Med 2013;1:621–9.
[16] Van Dyck L, Casaer MP, Gunst J. Autophagy and its implications against early full nutrition support in critical illness. Nutr Clin Pract 2018;33:339–47.
[17] Casaer MP. Muscle weakness and nutrition therapy in ICU. Curr Opin Clin Nutr Metab Care 2015;18:162–8.
[18] Patel JJ, Rosenthal MD, Heyland DK. Intermittent versus continuous feeding in critically ill adults. Curr Opin Clin Nutr Metab Care 2018;21:116–20.
[19] Heyland DK, Wischmeyer PE. Does artificial nutrition improve outcome of critical illness? An alternative viewpoint!. Crit Care (London, England) 2013;17:324.
[20] Cherry-Bukowiec JR. Optimizing nutrition therapy to enhance mobility in critically ill patients. Crit Care Nurs Q 2013;36:28–36.
[21] Eikermann M, Latronico N. What is new in prevention of muscle weakness in critically ill patients? Intensive Care Med 2013;39:2200–3.
[22] Hermans G, Clerckx B, Vanhullebusch T, et al. Interobserver agreement of Medical Research Council sum-score and handgrip strength in the intensive care unit. Muscle Nerve 2012;45:18–25.
[23] Tzanis G, Vasileiadis I, Zervakis D, et al. Maximum inspiratory pressure, a surrogate parameter for the assessment of ICU-acquired weakness. BMC Anesthesiol 2011;11:14.
[24] Stevens RD, Marshall SA, Cornblath DR, et al. A framework for diagnosing and classifying intensive care unit-acquired weakness. Crit Care Med 2009;37(Suppl 10):S299–308.
[25] Orem Dorothea E. Nursing Concepts of Practice. St. Louis: Mosby; 2001.
[26] Mahoney FI, Barthel DW. Functional evaluation: the Barthel index. Md State Med J 1965;14:61–5.
[27] Sarker SJ, Rudd AG, Douiri A, et al. Comparison of 2 extended activities of daily living scales with the Barthel index and predictors of their outcomes: cohort study within the South London Stroke Register (SLSR). Stroke 2012;43:1362–9.
[28] Kondrup J, Rasmussen HH, Hamberg O, et al. Nutritional risk screening (NRS 2002): a new method based on an analysis of controlled clinical trials. Clin Nutr (Edinburgh, Scotland) 2003;22:321–36.
[29] Detsky AS, Baker JP, Mendelson RA, et al. Evaluating the accuracy of nutritional assessment techniques applied to hospitalized patients: methodology and comparisons. JPEN J Parenter Enteral Nutr 1984;8:153–9.
[30] Finfer S, Chittock DR, Su SY, et al. Intensive versus conventional glucose control in critically ill patients. N Engl J Med 2009;360:1283–97.
[31] Wiener RS, Wiener DC, Larson RJ. Benefits and risks of tight glucose control in critically ill adults: a meta-analysis. JAMA 2008;300:933–44.
[32] Heyland DK, Day A, Clarke GJ, et al. Nutrition and Exercise in Critical Illness Trial (NEXIS Trial): a protocol of a multicentred, randomised controlled trial of combined cycle ergometry and amino acid supplementation commenced early during critical illness. BMJ Open 2019;9:e027893.
[33] Vincent JL, Moreno R, Takala J, et al. The SOFA (sepsis-related organ failure assessment) score to describe organ dysfunction/failure. On behalf of the Working Group on Sepsis-Related Problems of the European Society of Intensive Care Medicine. Intensive Care Med 1996;22:707–10.
[34] Puthucheary ZA, Rawal J, McPhail M, et al. Acute skeletal muscle wasting in critical illness. JAMA 2013;310:1591–600.
[35] Chen X, Yu R, Chen H. The effect of the bundle nursing on the prevention of intensive care unit acquired weakness in patients with mechanical ventilation. Zhejiang Clin Med J 2017;19:1733–4.
