# Supplementary material for: Effect of early mobilization combined with early nutrition on acquired weakness in critically ill patients (EMAS): A dual-center, randomized controlled trial
Source: PLoS One. 2022 May 26;17(5):e0268599. doi: 10.1371/journal.pone.0268599 (PMC9135241; doi:10.1371/journal.pone.0268599)
Supplement: S3 File — (PDF) [file pone.0268599.s004.pdf]

研究方案

项目名称：基于 Orem 自护理论的早期活动联合早期营养对危重症患者 ICU 获得性衰弱的干预效果研究

负责人：范宇莹

研究目的：研究以自护理论和 2019 年 ESPEN 指南为理论基础，构建基于自护理论和 2019 年 ESPEN 指南的 ICU 患者 ICU 获得性衰弱（intensive care unit-acquired weakness, ICU-AW）的干预方案，并采用定量与定性相结合的方法评价该模式的效果。

研究内容：（仅需描述涉及受试者拟开展的研究内容部分）

一、研究对象的确定

1、 抽样及分组方法

（1）抽样方法：采用便利取样的方法。具体操作方法：通过病例或面对面询问的方式，筛选出哈尔滨医科大学附属第二医院重症监护室初入ICU的住院患者，并以电话或面对面的方式告知患者或其代理人本研究的目的、意义等，在知情同意的前提下，将符合入组标准的患者纳入本研究。

（2）分组方法：用 Excel 进行简单随机分组。

2、样本量的确定

样本量由 PASS 11.0.7 软件计算得出。假定检验水准 α＝0.05（双侧），检验效能 β＝0.10（单侧），失访率取 10%，估计的效应大小值是基于转出 ICU 时 ICU-AW 发生率（早期活动组=33.1%，干预组=51.9%）[1,2]，软件计算得出至少需要 150 名研究对象（每组 50 名）。

3、纳入标准及排除标准

（1）纳入标准：①年龄≥18周岁；②首次入住ICU；③预计入住ICU时间≥72小时；④意识清楚，能清醒地回应以下简单命令中的至少3个：睁开或闭上眼睛，看着我，伸舌，点头，皱眉；⑤患者或其代理人知情同意。

（2）排除标准：①四肢残缺；②神经肌肉疾病，如重症肌无力、格林巴利综合征、肌萎缩侧索硬化；③影响运动功能的颅内或脊髓状态异常（如颅内压升高、脑卒中等）；④一个月内做过胃肠道手术；⑤患甲状腺功能亢进等影响营养和代谢的内分泌疾病；⑥怀孕；⑦癌症晚期、预后极差或短期内死亡；⑧在入住

ICU48小时内预计无法开展营养支持。

二、实验研究设计

本研究为随机对照实验研究，由研究者本人在 2020 年 5 月至 2021 年 4 月对重症监护室住院患者实施干预，旨在降低 ICU 患者 ICU 获得性衰弱发病率，并评价其干预后的效果。

（一）对照组干预措施

采取ICU常规护理：

①病情监测：严密监测生命体征、观察病情变化，定时测量CVP、动脉血压、血气分析，及时发现异常情况并处理；

②保持患者舒适：保持室内温度、湿度适宜，床单位整洁；

③做好各种管道护理；

④生活护理：口腔护理，会阴护理，二便护理，每2h翻身一次；

⑤康复运动：由康复治疗师对患者进行康复运动治疗，康复方式大部分均采取被动运动，无成形系统的运动方案；

⑥营养支持：遵医嘱启动营养支持，无成形的系统的方案。确认胃管或其他饲管位置妥当，床头抬高30~45°。监测是否出现胃肠综合症（腹泻、腹胀、腹痛、恶心或呕吐等），根据病人情况、反应调节喂养速度。进行运动时停止喂养，以防误吸。检测血糖与电解质。

三组除干预措施不同外，其余常规护理措施尽可能保持一致。均遵医嘱积极治疗原发疾病。除早期运动联合早期营养组，其他两组营养方案均为ICU常规营养方案。三组营养支持护理及实验室指标检测内容相同，给予营养速度均一致，营养物质成分也完全一致，能量消耗均使用体重权重方程20~25kcal/kg/d。

（二）早期运动组干预措施

在ICU常规护理的基础上，由康复治疗师、责任护士、研究者共同合作，在转入ICU治疗24h内，根据BI评分采取基于自护理论的早期运动干预策略，每日执行两次。该运动策略基于文献查阅和与专家咨询形成，为一种全新的循序渐进的科学的运动方案。具体干预措施如下：

①完全补偿系统：BI评分＜40分者，行四肢肌肉揉捏以及四肢关节被动活动。每个肢体各关节主要方向均重复10次。如上肢、手指的屈曲和伸展；腕关节的屈曲、伸展、桡偏和尺偏；肘关节的屈曲、伸展、外展、内收；肩关节的屈曲、外

展、内旋、外旋；

②部分补偿系统：BI评分≥40分且＜60分者，增加患者床头抬高角度至30-45°，被动活动每次每个肢体重复5次。双手握拳10s和踝泵运动15s，20个/次。给予床上坐位每次20分钟。可耐受者，于床上进行四肢关节主动活动，即上肢外展和扩胸运动、下肢行蹬腿运动，30个/次。每日辅助患者床旁坐位，每次20分钟；

③辅助教育系统：BI评分≥60分者，给予有关主动运动的健康教育，指导患者学会自主进行主动运动。在“部分补偿系统”中的主动运动内容的基础上，在康复师指导下协助患者靠床短时间站立5-10min。

当生命体征出现以下变化时 ，应暂停早期活动：心率＞130次/min或＜60次/min，休息时心率下降＞20%，心脏节律不规整；收缩压＞180mmHg（1mmHg=0.133kPa）或<90mmHg，平均动脉压＞100mmHg或＜60mmHg；血氧饱和度＜88%；呼吸频率＜5次/min或＞40次/min。机械通气者，呼吸机参数为氧浓度＞60%；PEEP＞10 cmH_2O（1 cmH_2O=0.098kPa），控制通气模式。

（三）早期运动联合早期营养组干预措施

ICU常规护理、早期运动的基础上，以2019年ESPEN指南[3]为指导，由ICU医生、责任护士、营养师共同合作，对早期运动联合早期营养组患者于48小时内开展早期营养方案。该方案基于国际最新的指南与专家咨询形成。具体早期营养方法如下：

NRS 2002评分≥3分且无营养支持禁忌症，能自主进食者首选经口喂养；不能自主进食者于48小时内开展早期持续性EN，外科术后患者24h内开展；严重营养不良患者（SGA-C）或存在高度营养不良风险者（NRS 2002评分≥5分）如存在EN禁忌症，则可早期提供渐进性少量PN。急性疾病早期阶段（三天内）提供热量不超过能量消耗的70%的低热量营养，三天后所给予的热量需增加至所测能量消耗值的80%-100%。能量消耗使用体重权重方程20~25kcal/kg/d。

三、评价

表-1. 资料收集内容及时间

资料	时间
患者的一般资料	入 ICU 24 小时内
BI 评分	入 ICU 24 小时内，干预开始后每 24 小时评估一次，所有干预结束后即即将转出 ICU 时

MRC-SS 评分	入 ICU 24 小时内，干预开始后每 24 小时评估一次，所有干预结束后
SOFA 评分	入 ICU 24 小时内，干预开始后每 24 小时评估一次，所有干预结束后
ICU 住院天数	所有干预结束后
机械通气时间	所有干预结束后
死亡人数	所有干预结束后

四、整理资料

资料收集完成后用SPSS（20.0）版进行统计分析，所有统计检验均采用双侧检验，检验水准α=0.05。

（1）对于患者的一般资料采用频数、百分比、均数或中位数进行描述，符合正态分布的内容用χ^2检验；

（2）对患者的BI评分、MRC-SS评分、SOFA评分、ICU住院天数、机械通气时间，应用均数和标准差或中位数和四分位数进行描述。

（3）患者ICU-AW发生率、死亡率用χ^2检验进行组间比较；

（4）三组不同时间点资料比较采用重复测量方差分析；

（5）针对患者BI评分、MRC-SS评分、SOFA评分、ICU住院天数、机械通气时间的组间比较，对于非正态分布资料采用非参数检验进行分析，符合正态分布资料采用*t*检验进行组间比较。

参考文献：

[1] Zang K, Chen B, Wang M, et al. The effect of early mobilization in critically ill patients: A meta-analysis[J]. Nurs Crit Care, 2020, 25(6): 360-367.

[2] Chen X, Yu R, Chen H. The effect of the bundle nursing on the prevention of intensive care unit acquired weakness in patients with mechanical ventilation[J]. Zhejiang Clinical Medical Journal, 2017, 19(9): 1733-1734.

[3] Singer P, Blaser AR, Berger MM, et al. ESPEN guideline on clinical nutrition in the intensive care unit[J]. Clin Nutr, 2019, 38(1): 48-79.
