# Supplementary material for: Effect of early mobilization combined with early nutrition on acquired weakness in critically ill patients (EMAS): A dual-center, randomized controlled trial
Source: PLoS One. 2022 May 26;17(5):e0268599. doi: 10.1371/journal.pone.0268599 (PMC9135241; doi:10.1371/journal.pone.0268599)
Supplement: S4 File — (PDF) [file pone.0268599.s005.pdf]

Study Protocol

Title: Effect of early mobilization combined with early nutrition on acquired weakness in ICU patients

Director: Yuying Fan

Purpose: This study adopted Orem self-care theory as the framework of early mobilization (EM) intervention, and European Society for Parental and Enteral Nutrition (ESPEN) guideline (2019) of early nutrition intervention, aiming to construct an innovative intervention strategy against intensive care unit-acquired weakness (ICU-AW), and to investigate whether the participation in early mobilization combined with early nutrition (EMN) program in the ICU is better than EM alone or routine ICU care.

Study content: (describe only the necessary part related to the implementation of the study)

1. Participants

1.1 Sampling and group allocation

1.1.1 Sampling

Convenient sampling method will be adopted. Through case books or face-to-face inquiry, patients admitted to ICUs of the Second Affiliated Hospital of Harbin Medical University and the First Affiliated Hospital of Jiamusi University will be screened, and the patients or their agents will be informed of the purpose and significance of this study by telephone or face-to-face. On the premise of informed consent, the patients who meet the inclusion criteria will be included in this study.

1.1.2 Group allocation

Simple random grouping with Excel will be applied in this trial.

1.2 Sample size calculation

With a power of 90% and 2-sided 5% level of significance, 49 participants per group (147 total) will be needed to detect a significant between-group difference in the primary outcome, as calculated by PASS (version 11.0.7) and allowing for up to 10% attrition at ICU discharge (i.e., 132 participants with complete data). The estimated effect size is based on the incidence of ICU-AW at ICU discharge (early mobilization group=33.1%, control group=51.9%) reported in a meta-analysis of early mobilization interventions for critically ill patients [1] as well as the findings of Chen (2017) in a clinical trial (15.9%) [2].

1.3 Inclusion and exclusion criteria.

1.3.1 Inclusion criteria

1) Age ≥18 years;
2) Admitted to ICU for the first time;
3) Expected ICU stay ≥72 hours;
4) Consciousness adequate to respond to at least three of these orders: "open/close your eyes", "look at me", "put out your tongue", "nod your head", "raise your eyebrows";
5) Informed consent of patients or their agents

1.3.2 Exclusion criteria

1) Incomplete limbs;
2) Pre-existing primary systemic neuromuscular disease (e.g., Guillain Barre,

myasthenia gravis, amyotrophic lateral sclerosis);

3) Intracranial or spinal process affecting motor function;
4) Had gastrointestinal surgery in one month;
5) Hyperthyroidism and other endocrine diseases affecting nutrition and metabolism;
6) Pregnant;
7) Has terminal cancer, expected death or extremely poor prognosis;
8) No expectation for any nutritional intake within the subsequent 48 hours

2. Study design and intervention

2.1 Study design

This is a randomized controlled trial conducted from May, 2020 to April 2021 for ICU patients, aiming at reduce the occurrence of ICU-AW.

2.2 Intervention

All three groups will receive standard ICU care, which includes conventional nutrition support and physiotherapy. With the exception of patients in the ENM group, all patients in the other two groups will receive conventional nutrition therapy. The contents of nutritional support nursing, laboratory index testing, feeding speed, nutritional targets and nutrient composition are completely consistent among the three groups. Nutritional targets will be measured by weight-based equation 20-25 kcal/kg/d.

2.2.1 Control group. Participants in the control group will only receive standard ICU care. The usual care strategy is as follows:

1. Closely monitor vital signs, observe any changes in illness; regularly measure

central venous pressure (CVP), arterial blood pressure and blood gas analysis; identify and manage abnormal conditions in a timely manner.

2. Maintain indoor temperature and humidity at suitable levels and keep the bed clean and tidy.
3. Carefully manage all types of tubes.
4. Perform oral nursing care, perineal care, bedsore nursing; turn patient over every 2 h.
5. Invite the occupational therapist, according to the ICU doctor's order, to perform rehabilitation exercise therapy for certain patients with no fixed time, method, or frequency, as described above. The present conventional methods include massaging muscles; passive, active assisted, or active mobilization; and bed positioning and maintenance of orthostatism. Nevertheless, the type of therapy will not be defined in advance, but instead will be at the discretion of the attending physiotherapist and will not have a pre-established routine.
6. Provide nutritional support as ordered by the doctors. Energy requirements are calculated as 20 to 25 kcal/kg/day. Different doctors have different strategies of nutrition support, such as initiation time, route of nutrition, and inclusion or exclusion criteria for nutrition therapy.

2.2.2 Early mobilization group. Patients in the EM group will receive early mobilization intervention twice daily in addition to standard treatment. Physiotherapists, nurses responsible for the patients, and the study researchers will collaborate to implement early mobilization intervention based on the patient's Barthel Index (BI) score starting within 24 h of ICU admission, twice daily. The early

mobilization intervention is created by the research team based on data in the academic literature.

Mode 1: Wholly compensatory system

BI score: <40.

Exercises: Passive movement. Perform muscle kneading and passive movement of extremities twice per day. Exercise in the main direction of the joints of each limb will be repeated 10 times, such as flexion and extension of upper limbs and fingers; flexion, extension, radial deviation and ulnar deviation of the wrist joint; flexion, extension, abduction and adduction of the elbow joint; and flexion, abduction, internal rotation and external rotation of the shoulder joint.

Mode 2: Partly compensatory system

BI score: ≥40 and <60.

Exercises: Passive movement combined with active movement. The bed-head angle will be increased to 30-45°, and the passive exercise described in mode one will be repeated 5 times with each limb. Clenching fists for 10 s and the ankle pump exercise for 15 s will both be performed 20 times per side. Active sitting in bed will be performed for 20 min. If the patient is able to finish the above exercises, he or she will perform active movement of the joints of the extremities while on the bed; that is, chest expansion and abduction of the upper limbs (arms) and kicking of the lower limbs (legs), all repeated 30 times. Patient will then perform assisted bedside sitting for 20 min. Patients capable of these movements will progress to assisted standing against the bed for 5 min.

Mode 3: Supportive-educative system

BI score: ≥60.

Exercises: Active movement. Patient will perform active bedside sitting for 10 min, and active standing against the bed for 10 min. Patients who can achieve these will shift to standing independently for 5 min, then marking time for 10 min.

The therapy session will be paused or terminated if the patient:

- Has a heart rate above 130 bpm/min or below 60 bpm/min
- Has a heart rate decreasing by more than 20% while resting, with irregular rhythm
- Has a systolic blood pressure above 180 mmHg or below 90 mmHg, or mean arterial pressure above 100 mmHg or below 60 mmHg
- Has a blood oxygen saturation below 88%
- Has a respiratory rate below 5 breaths/min or above 40 breaths/min
- Receives mechanical ventilation, and the oxygen concentration is above 60%; or the positive end expiratory pressure (PEEP) is above 10 cmH_2O, and the patient is ventilated by control mode (CMV)

2.2.3 Early mobilization combined with early nutrition group. In addition to ICU routine nursing and the early mobilization intervention, patients in the ENM group will receive an early medical nutrition program based on the 2018 European Society for Clinical Nutrition and Metabolism (ESPEN) guideline [13] and the academic literature; this will be initiated within 48 h of ICU admission.

Patients with a Nutritional risk screening (NRS 2002) score ≥3, which indicates the need for nutrition support [3], will receive early medical nutrition therapy consisting of early oral nutritional supplements and artificial nutrition (enteral

nutrition, parenteral nutrition) [4]. Oral feeding is preferred over artificial nutrition when the patient is able to eat independently. Patients who cannot take food by mouth will be given early persistent EN within 48 h. Postoperative patients will be supplied with EN within 24 h after surgery. Early enteral nutrition (EN) is preferred over early parenteral nutrition (PN). Patients with severe malnutrition (grade C of the subjective global assessment (SGA)) [5] or with high nutrition risk (NRS 2002 score ≥5) [3] will receive early progressive low-dose parenteral nutrition (PN) if they have EN contraindications. At the early stage of the acute phase of injury (within three days), patients will be provided with low-calorie nutrition of no more than 70% of their measured energy consumption. After three days, the amount of calories given should be increased to 80% of the measured energy consumption. Patients allocated to the ENM group will have energy requirements calculated as 20 to 25 kcal/kg/day.

3. Outcomes

Table 1. Outcome assessment.

Outcomes	Timepoint
Baseline characteristics	Enrolment
BI	Enrolment, every 24 hours following enrollment, ICU discharge
MRC sum-score	Enrolment, ICU discharge
SOFA score	Enrolment, every 24 hours following enrollment, ICU discharge
Time of ICU stay	ICU discharge
Duration of mechanical ventilation	ICU discharge
ICU mortality	ICU discharge

BI = Barthel index, ICU = intensive care unit, ICU-AW = intensive care unit-acquired weakness, MRC = Medical Research Council, SOFA = Sequential Organ Failure Assessment.

4. Statistical analysis

Statistical analyses will be conducted by an independent statistician using SPSS Statistics software (version 20.0) adopting two-tailed tests, with *p* values below 5%

indicating statistical significance.

Continuous data were expressed as mean ± standard deviation (normal variables) or medians (non-normal variables), and categorical data were presented as number (%). Normal variables among three groups were compared by one-way ANOVA analysis, non-normal variables and ranked data with nonparametric Kruskal Wallis test, and categorical data (except for ranked data) as well as their pairwise comparisons with Chi-squared test. The data of three groups at different time points will be compared by repeated measurement analysis of variance.

Reference：

1. Zang K, Chen B, Wang M, Chen D, Hui L, Guo S, et al. The effect of early mobilization in critically ill patients: A meta-analysis. Nursing in critical care. 2019:1-8.

2. Chen X, Yu R, Chen H. The effect of the bundle nursing on the prevention of intensive care unit acquired weakness in patients with mechanical ventilation. Zhejiang Clinical Medical Journal. 2017;19(9):1733-4.

3. Kondrup J, Rasmussen HH, Hamberg O, Stanga Z. Nutritional risk screening (NRS 2002): a new method based on an analysis of controlled clinical trials. Clinical nutrition (Edinburgh, Scotland). 2003;22(3):321-36.

4. Singer P, Blaser AR, Berger MM, Alhazzani W, Calder PC, Casaer MP, et al. ESPEN guideline on clinical nutrition in the intensive care unit. Clinical nutrition (Edinburgh, Scotland). 2019;38(1):48-79.

5. Detsky AS, Baker JP, Mendelson RA, Wolman SL, Wesson DE, Jeejeebhoy KN.

Evaluating the accuracy of nutritional assessment techniques applied to hospitalized patients: methodology and comparisons. JPEN Journal of parenteral and enteral nutrition. 1984;8(2):153-9.
