# Supplementary material for: Effect of early mobilization combined with early nutrition on acquired weakness in critically ill patients (EMAS): A dual-center, randomized controlled trial
Source: PLoS One. 2022 May 26;17(5):e0268599. doi: 10.1371/journal.pone.0268599 (PMC9135241; doi:10.1371/journal.pone.0268599)
Supplement: S5 File — (PDF) [file pone.0268599.s006.pdf]

Clinical triage documentation

Order:________

Group:________

Patient ID:________

ICU diagnose:________

Age:________

BMI:________

APACHE II score:________

Education level:________

High risk factors of ICU-AW:________

Barthel Index (14 d before ICU admission):________

NRS 2002 score:________

SOFA score:________

Medical insurance (no/yes):________

MV (no/yes):________
