# Supplementary material for: Effect of early mobilization combined with early nutrition on acquired weakness in critically ill patients (EMAS): A dual-center, randomized controlled trial
Source: PLoS One. 2022 May 26;17(5):e0268599. doi: 10.1371/journal.pone.0268599 (PMC9135241; doi:10.1371/journal.pone.0268599)
Supplement: S1 Data — (PDF) [file pone.0268599.s007.pdf]

Order	Group	ID	Gender	Age	Weight	Height	BMI	APACHE Ⅱ score	Education level	Diagnosis	High risk factors of ICU-AW	MRC			BI			NRS 2002	SGA.123		△SOFA				ICU stay	Medical insurance	MV		Death	ICU stay
			M1F0	(yr)	(kg)	(m)	(kg/m^2)		1Primary school or less<br>2Middle school<br>3High school<br>4College or above	1Renal failure<br>2Gastrointestinal/hepatic disorder<br>3Respiratory disorder<br>4Acute pancreatitis<br>5Cardiac disorder<br>6Infection	1Diabetes<br>2Infection<br>3Post-surgery<br>4None<br>5Two factors combined<br>6Three factors combined	Before	After	AW	2W before	Before	After		Before	After	Before	After	Max	△	(d)		(h)			(d)
**1**	ENM	2213511	1	50	70	1.6	27.34	18	4	1	2	52	60	0	100	60	70	1	1	1	10	6	10	0	5.13	0	0.00	0	0	5.13
**2**	EM	2150510	0	52	75	1.6	29.30	26	1	2	4	49	46	1	100	25	45	3	2	2	16	10	19	3	9.71	0	58.00	1	0	9.71
**3**	ENM	2214051	1	76	63.5	1.68	22.50	22	1	3	4	42	42	1	95	10	10	3	2	2	10	12	12	2	2.27	1	54.55	1	1	2.27
**4**	control	2213512	0	24	85	1.65	31.22	4	3	2	5	60	60	0	100	75	65	1	1	1	8	2	8	0	3.25	0	0.00	0	0	3.25
**5**	control	2216194	0	67	55	1.5	24.44	16	2	7	1	60	60	0	100	35	45	3	1	1	6	6	8	2	3.40	1	0.00	0	0	3.40
**6**	ENM	2211594	0	74	43	1.53	18.37	17	1	3	4	54	60	0	100	60	70	4	3	2	7	0	7	0	1.53	0	16.22	1	0	1.53
**7**	EM	2218216	1	76	65	1.7	22.49	12	2	7	1	40	48	0	100	5	10	3	1	1	8	12	12	4	12.88	0	152.00	1	1	12.88
**8**	control	2217106	1	68	75	1.72	25.35	22	1	3	5	54	57	0	100	35	40	2	1	1	4	4	5	1	3.04	0	35.00	1	0	3.04
**9**	EM	2219256	0	55	50	1.59	19.78	9	3	6	2	49	60	0	100	20	50	1	1	1	6	7	9	3	7.56	1	37.00	1	0	7.56
**10**	ENM	2217232	0	66	86	1.7	29.76	17	4	1	2	60	60	0	100	65	70	6	3	1	6	2	6	0	3.17	1	0.00	0	0	3.17
**11**	control	2215522	1	65	75	1.7	25.95	10	2	5	3	60	60	0	100	55	50	2	1	1	6	4	6	0	6.04	1	18.50	1	0	6.04
**12**	control	2214803	1	70	62	1.57	25.15	12	1	5	3	45	55	0	100	5	35	4	1	2	5	6	10	5	8.85	1	141.00	1	0	8.85
**13**	EM	2221644	1	70	60	1.65	22.04	19	3	1	4	60	60	0	100	65	85	4	2	1	11	4	11	0	3.46	1	0.00	0	0	3.46
**14**	EM	2219799	1	79	50	1.6	19.53	16	4	3	2	58	58	0	80	45	45	5	1	1	6	6	6	0	2.67	1	0.00	0	0	2.67
**15**	ENM	2057887	1	60	70	1.75	22.86	13	3	7	6	60	60	0	100	35	55	4	2	2	2	1	4	2	3.25	1	77.00	1	0	3.25
**16**	EM	2213036	0	77	40	1.55	16.65	18	3	2	4	60	60	0	70	55	70	5	3	2	7	3	7	0	5.59	0	0.00	0	0	5.59
**17**	EM	2225638	0	54	62	1.55	25.81	10	3	3	5	54	56	0	100	20	70	2	1	1	3	1	3	0	4.65	1	58.50	1	0	4.65
**18**	ENM	2225603	0	64	47	1.55	19.56	12	1	6	5	60	60	0	100	40	94	4	1	1	4	0	5	1	4.60	1	119.00	1	0	4.60
**19**	control	2226037	1	50	85	1.77	27.13	4	4	3	5	60	60	0	100	85	70	2	1	1	3	2	7	4	12.92	1	0.00	0	0	12.92
**20**	control	2226063	0	63	62.5	1.6	24.41	12	3	1	4	60	60	0	100	60	65	5	3	2	6	5	6	0	7.10	1	0.00	0	0	7.10
**21**	ENM	2227184	0	61	60	1.59	23.73	7	3	6	6	60	60	0	100	70	85	4	3	1	3	1	3	0	3.83	0	18.00	1	0	3.83
**22**	EM	2227178	0	60	77	1.66	27.94	11	1	1	4	60	60	0	100	55	85	4	2	1	9	5	9	0	5.52	1	0.00	0	0	5.52
**23**	ENM	2228772	0	64	65	1.6	25.39	21	3	3	1	58	50	0	100	20	35	5	2	2	9	4	13	4	9.58	1	230.00	1	0	9.58
**24**	ENM	2227756	1	69	88	1.82	26.57	16	2	1	4	60	60	0	100	60	60	5	3	3	6	9	9	3	1.99	1	0.00	0	0	1.99
**25**	control	2228833	0	62	50	1.6	19.53	12	2	7	2	57	35	1	95	60	15	6	3	3	6	9	9	3	3.13	1	21.00	1	0	3.13
**26**	EM	2230431	0	60	57	1.58	22.83	18	1	7	4	60	59	0	100	5	65	4	1	1	6	4	8	2	12.25	0	114.50	1	0	12.25
**27**	EM	2230460	0	22	70	1.65	25.71	9	4	4	4	60	60	0	100	65	70	3	2	2	0	2	3	3	3.44	0	0.00	0	0	3.44
**28**	EM	2230444	1	83	95	1.78	29.98	18	2	5	1	58	58	0	95	5	5	7	3	3	3	3	3	0	1.84	0	0.00	0	0	1.84
**29**	control	2231638	1	48	105	1.65	38.57	9	2	6	6	60	60	0	100	70	70	3	1	1	9	2	9	0	3.02	1	69.50	1	0	3.02
**30**	EM	2235403	1	67	60	1.65	22.04	12	2	2	5	60	60	0	100	60	75	4	2	2	10	7	12	2	4.79	1	0.00	0	0	4.79
**31**	control	2236601	1	77	60	1.7	20.76	15	2	2	2	54	35	1	95	35	10	6	2	2	8	11	11	3	4.04	1	0.00	0	0	4.04
**32**	EM	2238820	0	67	60	1.6	23.44	23	1	1	1	60	60	0	100	55	55	5	3	3	7	7	7	0	1.27	1	0.00	0	0	1.27
**33**	EM	2025778	1	64	102.5	1.7	35.47	8	1	2	1	60	60	0	95	75	75	3	2	2	6	6	6	0	1.62	0	0.00	0	0	1.62
**34**	ENM	2238679	0	52	40	1.56	16.44	13	2	3	4	57	57	0	100	70	70	6	2	2	4	4	4	0	1.92	1	46.17	1	0	1.92
**35**	control	2240297	0	55	62	1.62	23.62	13	2	1	5	55	58	0	100	50	45	6	3	1	7	4	10	3	11.22	1	206.33	1	0	11.22
**36**	control	2237321	0	57	65	1.64	24.17	10	2	5	3	56	64	0	100	10	40	1	1	1	5	4	10	5	4.96	1	119.00	1	0	4.96
**37**	ENM	2241232	1	43	70	1.64	26.03	11	1	3	2	60	60	0	95	75	80	5	2	1	3	6	6	3	4.05	1	0.00	0	0	4.05
**38**	control	2242073	0	59	55	1.6	21.48	19	2	3	2	60	60	0	100	40	40	3	1	1	9	8	9	0	2.69	0	42.25	1	0	2.69
**39**	EM	2241950	0	45	60	1.67	21.51	11	2	2	2	60	60	0	100	60	40	5	2	1	6	1	12	6	7.15	0	0.00	0	0	7.15
**40**	EM	2243448	1	77	85	1.77	27.13	15	4	3	2	60	60	0	95	50	50	4	2	2	7	7	7	0	1.98	0	47.50	1	0	1.98
**41**	ENM	2244079	0	68	55	1.55	22.89	12	2	4	5	60	60	0	100	50	40	6	2	2	5	7	7	2	3.73	0	2.17	1	0	3.73
**42**	control	2206297	1	49	72	1.74	23.78	13	1	1	4	60	60	0	100	45	10	5	3	3	12	8	12	0	6.13	1	0.00	0	0	6.13
**43**	EM	2245270	1	44	57.5	1.71	19.66	8	3	4	4	59	60	0	100	55	80	3	2	2	8	5	10	2	11.75	0	84.42	1	0	11.75
**44**	EM	2245547	1	58	55	1.65	20.20	10	1	6	4	60	55	0	100	65	55	3	3	2	6	4	6	0	4.35	0	0.00	0	0	4.35
**45**	ENM	1839723	1	66	60	1.67	21.51	20	2	2	1	60	60	0	95	10	55	5	3	2	4	9	17	13	11.17	1	163.00	1	0	11.17
**46**	ENM	2245577	1	68	70	1.7	24.22	8	3	7	4	60	60	0	100	60	60	1	1	1	4	4	4	0	1.86	0	0.00	0	0	1.86
**47**	control	2205833	0	65	40	1.56	16.44	19	3	7	1	37	26	1	95	20	20	6	3	3	6	6	7	1	3.05	1	0.00	0	0	3.05
**48**	ENM	2116454	1	34	83	1.83	24.78	8	4	1	3	60	60	0	100	50	65	4	3	2	7	4	7	0	3.01	1	0.00	0	0	3.01
**49**	ENM	2247106	0	65	55	1.65	20.20	20	1	5	4	54	58	0	100	25	40	5	2	2	8	16	16	8	3.00	0	62.00	1	0	3.00
**50**	control	2247717	1	60	60	1.73	20.05	13	2	1	5	60	46	1	100	75	30	6	3	3	12	11	13	1	4.56	0	0.00	0	0	4.56
**51**	control	2248389	1	51	55	1.72	18.59	12	2	4	4	60	60	0	100	65	80	6	3	3	5	6	7	2	11.02	0	0.00	0	0	11.02
**52**	EM	2248502	1	34	83	1.75	27.10	1	2	2	2	60	60	0	100	90	100	3	2	1	4	5	6	2	3.96	0	0.00	0	0	3.96
**53**	control	2250013	1	42	78	1.72	26.37	11	3	2	1	60	60	0	100	68	80	6	2	2	7	3	7	0	6.01	1	0.00	0	0	6.01
**54**	ENM	2251179	1	74	65	1.78	20.52	11	3	6	5	58	58	0	100	60	60	5	2	2	4	2	7	3	6.73	1	137.00	1	0	6.73
**55**	EM	2250983	0	72	60	1.58	24.03	15	3	3	4	57	60	0	100	15	40	7	1	1	5	5	6	1	9.26	1	261.38	1	0	9.26
**56**	control	2251648	1	62	67.5	1.73	22.55	10	1	7	4	60	60	0	100	45	60	2	2	2	4	7	7	3	3.01	1	0.00	0	0	3.01
**57**	EM	2252288	0	29	60	1.68	21.26	9	5	7	4	60	60	0	100	30	85	2	1	1	1	0	1	0	3.01	0	0.00	0	0	3.01
**58**	EM	2253687	0	47	54.5	1.58	21.83	13	1	2	4	55	58	0	100	55	60	5	2	1	1	3	3	2	10.48	0	0.00	0	0	10.48
**59**	EM	2250718	1	68	65	1.66	23.59	16	2	7	3	54	57	0	100	35	60	3	1	2	5	5	9	4	10.80	0	214.83	1	0	10.80
**60**	control	2254116	1	66	80	1.73	26.73	24	3	3	2	60	50	0	100	25	15	4	1	2	10	10	15	5	7.01	0	174.00	1	0	7.01

**61**	control	2230722	1	72	85	1.76	27.44	8	3	5	5	60	60	0	100	20	80	5	2	1	4	3	4	0	4.57	1	0.00	0	0	4.57
**62**	ENM	2254804	1	70	76	1.8	23.46	29	2	3	2	43	49	0	70	35	35	7	3	3	9	10	12	3	4.82	0	55.67	1	0	4.82
**63**	ENM	2254880	1	69	85	1.75	27.76	19	3	1	1	60	58	0	100	25	45	6	3	2	7	5	8	1	9.31	0	59.00	1	0	9.31
**64**	EM	2254579	1	57	75	1.8	23.15	19	2	2	1	60	60	0	70	55	60	6	3	2	6	4	6	0	3.01	0	0.00	0	0	3.01
**65**	control	2246043	1	63	85	1.77	27.13	15	3	5	5	59	55	0	85	45	10	6	3	3	10	18	18	8	5.80	1	112.50	1	0	5.80
**66**	ENM	2255621	0	66	75	1.58	30.04	22	2	7	1	60	60	0	95	40	55	3	2	2	7	6	7	0	3.01	0	19.50	1	0	3.01
**67**	EM	2256428	0	19	55	1.53	23.50	17	1	7	2	47	60	0	100	25	100	6	3	1	1	0	1	0	5.58	0	0.00	0	0	5.58
**68**	EM	2256474	1	28	115	1.9	31.86	7	2	7	4	60	60	0	100	90	95	1	3	1	10	3	10	0	3.01	0	0.00	0	0	3.01
**69**	EM	2256897	1	47	80	1.68	28.34	18	3	4	2	59	60	0	100	60	60	6	2	2	11	9	12	1	14.01	0	117.17	1	1	14.01
**70**	ENM	2257689	1	54	60	1.68	21.26	10	2	3	4	60	60	0	100	70	90	4	2	1	6	2	6	0	3.01	0	0.00	0	0	3.01
**71**	ENM	2257410	0	78	62	1.58	24.84	30	1	3	5	60	60	0	90	35	70	7	2	1	10	3	10	0	3.01	0	40.00	1	0	3.01
**72**	control	2258922	0	79	50	1.52	21.64	26	2	3	2	40	35	1	85	25	10	7	3	3	10	13	16	6	3.44	1	40.08	1	0	3.44
**73**	ENM	2259444	0	81	55	1.65	20.20	14	1	3	5	60	60	0	100	60	60	6	2	2	1	3	4	3	3.00	1	67.67	1	0	3.00
**74**	EM	2258873	0	62	65	1.6	25.39	12	3	6	2	60	60	0	100	45	40	6	3	3	10	14	14	4	1.62	0	22.73	1	1	1.62
**75**	EM	2250489	1	58	75	1.76	24.21	16	3	7	2	60	60	0	100	60	80	3	1	1	6	4	7	1	12.06	1	0.00	0	0	12.06
**76**	EM	2256820	1	59	69	1.82	20.83	21	4	3	2	60	60	0	85	70	75	4	1	1	4	3	4	0	3.14	0	0.58	1	0	3.14
**77**	control	2260407	1	45	73	1.81	22.28	19	2	3	5	60	60	0	100	90	70	6	2	1	6	1	6	0	4.03	1	0.00	0	0	4.03
**78**	ENM	2260453	0	68	45	1.52	19.48	17	1	5	4	60	60	0	100	65	90	5	1	1	7	2	7	0	3.00	0	45.75	1	0	3.00
**79**	ENM	2219810	0	58	70	1.6	27.34	21	1	7	5	60	60	0	100	75	80	5	2	1	6	6	6	0	3.68	0	0.00	0	0	3.68
**80**	ENM	2258598	0	25	59.5	1.66	21.59	22	2	7	4	60	60	0	100	75	95	3	1	1	8	3	8	0	8.15	1	79.33	1	0	8.15
**81**	control	2262978	1	43	70	1.8	21.60	16	2	1	4	60	60	0	100	70	60	4	3	1	5	5	7	2	3.57	0	0.00	0	0	3.57
**82**	control	1943301	0	30	65	1.62	24.77	6	2	4	4	60	60	0	100	70	75	3	3	2	1	2	2	1	3.17	0	0.00	0	0	3.17
**83**	ENM	2111239	0	55	40	1.62	15.24	13	4	7	2	60	60	0	90	70	45	6	3	2	7	7	8	1	6.14	1	99.78	1	0	6.14
**84**	control	2263575	0	65	44	1.52	19.04	10	1	7	4	60	60	0	100	65	70	3	2	1	4	4	4	0	3.62	0	0.00	0	0	3.62
**85**	EM	2264253	0	64	50.5	1.56	20.75	13	2	3	2	60	60	0	100	60	70	3	1	2	2	3	5	3	7.56	0	180.53	1	0	7.56
**86**	control	2264332	0	79	79	1.58	31.65	8	3	3	2	60	47	1	100	65	40	4	2	2	3	8	8	5	4.21	0	100.93	1	0	4.21
**87**	control	2264209	1	46	86	1.82	25.96	30	4	1	1	46	40	1	100	10	0	5	3	3	10	6	17	7	7.79	1	159.12	1	0	7.79
**88**	control	2265176	0	40	52.5	1.62	20.00	10	1	7	4	60	60	0	100	20	55	3	3	2	8	4	8	0	3.05	0	0.00	0	0	3.05
**89**	control	2265854	1	41	70	1.65	25.71	25	2	4	2	60	47	1	100	35	0	5	3	3	5	14	20	15	5.69	0	184.48	1	0	5.69
**90**	EM	2266007	1	72	69	1.69	24.16	19	1	7	4	60	60	0	100	60	75	7	3	1	6	2	6	0	3.68	1	0.00	0	0	3.68
**91**	control	2256395	1	47	85	1.73	28.40	6	2	7	5	60	60	0	100	45	45	6	2	2	2	3	3	1	3.73	1	0.00	0	0	3.73
**92**	ENM	2266445	1	55	85	1.66	30.85	12	2	2	4	60	60	0	85	60	75	6	3	1	9	5	9	0	3.79	0	0.00	0	0	3.79
**93**	EM	2280681	1	86	75	1.65	27.55	19	3	3	4	58	56	0	90	40	5	5	1	2	6	6	8	2	4.35	0	103.00	1	0	4.35
**94**	EM	2257337	0	47	55	1.65	20.20	15	2	7	2	54	57	0	100	45	50	3	2	2	5	9	9	4	8.21	0	101.83	1	0	8.21
**95**	EM	2281965	1	55	86.5	1.73	28.90	16	4	4	1	60	60	0	100	60	65	5	3	1	6	5	6	0	3.01	0	0.00	0	0	3.01
**96**	control	2282327	0	59	52	1.55	21.64	25	1	1	4	59	60	0	100	45	80	4	1	3	7	2	7	0	3.01	0	0.00	0	0	3.01
**97**	ENM	2278366	1	80	55	1.70	19.03	20	2	5	4	52	55	0	100	55	55	7	3	2	12	13	13	1	3.00	1	0.00	0	0	3.00
**98**	control	2283723	1	66	65	1.70	22.49	12	2	2	2	60	60	0	100	40	60	5	3	2	12	11	12	0	3.03	0	0.00	0	0	3.03
**99**	ENM	2283729	1	85	65	1.72	21.97	19	1	2	2	45	58	0	100	40	60	6	3	2	9	5	9	0	6.39	1	0.00	0	0	6.39
**100**	control	2284945	1	52	72	1.70	24.91	8	3	6	2	60	60	0	100	60	70	2	2	2	12	8	12	0	4.02	0	0.00	0	0	4.02
**101**	ENM	2285442	1	44	58	1.75	18.94	17	1	6	2	60	60	0	90	40	40	4	2	2	11	11	11	0	1.15	1	27.50	1	0	1.15
**102**	EM	2285338	0	49	57	1.55	23.73	20	2	7	4	60	60	0	85	65	0	6	3	3	3	4	8	5	3.01	0	38.00	1	0	3.01
**103**	control	2284622	1	33	75	1.75	24.49	20	2	5	3	60	60	0	100	60	70	3	1	1	7	5	7	0	3.02	0	0.00	0	0	3.02
**104**	EM	2278685	1	58	75	1.80	23.15	6	4	2	1	60	60	0	100	95	100	1	1	1	5	5	5	0	3.00	1	0.00	0	0	3.00
**105**	ENM	2243951	0	66	65	1.58	26.04	20	1	1	1	60	60	0	75	65	80	6	2	1	4	3	4	0	3.00	0	0.00	0	0	3.00
**106**	control	2286116	0	60	63.5	1.61	24.50	9	1	4	4	60	60	0	100	60	60	3	3	2	4	3	4	0	5.35	0	0.00	0	0	5.35
**107**	ENM	2287215	1	46	80	1.80	24.69	21	3	1	1	28	60	0	100	5	80	6	2	2	9	7	11	2	6.58	1	110.55	1	0	6.58
**108**	ENM	2287435	0	57	62	1.65	22.77	11	2	7	4	60	60	0	100	50	90	5	2	1	1	0	1	0	7.36	0	0.00	0	0	7.36
**109**	ENM	2288188	0	31	40	1.60	15.63	8	1	2	3	60	60	0	90	75	75	4	3	2	2	0	2	0	3.00	0	15.00	1	0	3.00
**110**	control	2288293	1	52	55	1.65	20.20	10	1	2	4	60	60	0	100	65	85	1	2	1	5	4	5	0	3.00	0	0.00	0	0	3.00
**111**	EM	2289335	0	53	65	1.60	25.39	20	2	4	4	60	60	0	100	55	60	6	3	2	5	7	15	10	16.01	1	287.95	1	0	16.01
**112**	control	2285451	1	44	82	1.75	26.78	8	2	5	3	39	60	0	100	25	70	3	3	2	9	6	9	0	4.58	1	16.67	1	0	4.58
**113**	EM	2289902	0	42	62.5	1.64	23.24	6	1	7	4	60	60	0	100	65	75	0	1	1	3	2	3	0	3.00	1	72.03	1	0	3.00
**114**	control	2288747	1	67	70	1.65	25.71	17	2	7	1	60	60	0	100	40	85	3	1	1	2	2	2	0	4.16	1	0.00	0	0	4.16
**115**	ENM	2290436	1	61	110	1.80	33.95	14	1	7	2	60	60	0	100	50	75	4	1	1	5	4	6	1	3.00	0	0.00	0	0	3.00
**116**	ENM	2287770	1	47	64	1.78	20.20	18	4	6	5	57	60	0	100	55	70	5	2	1	2	3	3	1	4.73	0	0.00	0	0	4.73
**117**	control	2290082	0	71	45	1.56	18.49	19	1	1	4	56	59	0	90	60	55	7	3	3	8	4	8	0	5.12	1	0.00	0	1	5.12
**118**	ENM	2295338	0	64	65	1.55	27.06	11	1	3	2	60	60	0	95	45	0	4	2	1	3	7	8	5	3.65	0	51.12	1	1	3.65
**119**	ENM	2295694	1	33	83	1.76	26.79	18	4	6	2	60	60	0	100	60	60	6	3	2	8	6	8	0	3.85	1	0.00	0	0	3.85
**120**	ENM	2296039	1	64	63	1.70	21.80	11	2	1	5	60	60	0	100	85	95	3	2	1	4	3	4	0	3.08	1	0.00	0	0	3.08
**121**	ENM	2295983	0	73	55	1.68	19.49	21	3	1	2	60	60	0	100	65	70	5	3	1	7	4	7	0	3.01	0	0.00	0	0	3.01
**122**	ENM	2296109	0	47	65	1.68	23.03	22	1	1	4	54	60	0	100	35	65	6	3	1	11	5	11	0	8.21	0	0.00	0	0	8.21
**123**	control	2298310	0	58	40	1.50	17.78	22	1	7	1	54	58	0	100	40	50	6	3	2	4	6	4	0	5.72	0	42.13	1	0	5.72
**124**	EM	2122698	1	67	69.8	1.67	25.03	14	3	5	5	60	60	0	100	60	60	5	2	1	7	11	11	4	3.47	1	6.33	1	0	3.47
**125**	control	2292563	0	79	55	1.65	20.20	23	4	3	4	60	60	0	100	65	65	5	2	2	3	3	6	3	7.25	1	173.92	1	1	7.25
**126**	EM	2301873	1	52	70	1.78	22.09	15	4	6	5	60	60	0	95	60	80	4	2	1	7	4	7	0	5.68	0	97.38	1	0	5.68
**127**	EM	2301878	1	26	75	1.77	23.94	20	4	4	4	60	60	0	100	50	90	6	3	1	3	1	4	1	15.01	0	0.00	0	0	15.01

**128**	EM	2243375	1	50	75	1.70	25.95	7	2	3	1	60	60	0	100	75	85	1	1	1	4	3	4	0	4.86	1	0.00	0	0	4.86
**129**	ENM	2303249	0	26	64	1.68	22.68	16	4	3	2	60	60	0	100	80	85	4	2	1	6	4	7	1	4.42	0	0.00	0	0	4.42
**130**	ENM	2297452	0	63	52	1.65	19.10	13	2	1	2	60	60	0	100	30	70	4	2	1	6	3	6	0	3.01	1	0.00	0	0	3.01
**131**	ENM	2307499	0	24	44	1.58	17.63	19	4	7	4	60	60	0	100	85	90	4	2	1	2	1	2	0	3.55	0	0.00	0	0	3.55
**132**	ENM	2307531	0	52	97	1.65	35.63	13	2	1	4	60	60	0	100	60	75	5	2	1	5	7	9	4	4.57	0	0.00	0	0	4.57
**133**	control	2306839	1	58	52.5	1.73	17.54	5	5	2	4	60	60	0	100	30	70	6	3	2	3	2	3	0	3.64	0	0.00	0	0	3.64
**134**	EM	2307473	0	67	70	1.65	25.71	20	1	2	1	60	60	0	85	30	75	6	3	2	10	5	10	0	3.01	0	0.00	0	0	3.01
**135**	control	2273820	1	35	69	1.68	24.45	19	2	1	4	60	60	0	100	80	80	4	3	2	6	5	6	0	4.63	1	0.00	0	0	4.63
**136**	ENM	2310788	1	51	83	1.71	28.38	7	2	4	4	60	60	0	100	75	85	3	3	2	3	0	3	0	3.01	0	0.00	0	0	3.01
**137**	ENM	2306917	1	52	64	1.68	22.68	17	4	5	1	60	60	0	100	40	60	4	2	1	7	4	7	0	3.00	1	0.00	0	0	3.00
**138**	control	2304234	1	67	72	1.69	25.21	14	2	5	3	60	60	0	100	50	55	4	2	2	5	4	5	0	3.76	1	0.00	0	0	3.76
**139**	EM	2310848	1	62	80	1.60	31.25	13	2	7	3	60	60	0	100	70	75	4	2	2	6	5	6	0	3.00	0	0.00	0	0	3.00
**140**	control	2311325	0	53	65	1.62	24.77	12	1	7	4	60	60	0	100	70	70	4	2	2	4	3	4	0	3.50	1	0.00	0	0	3.50
**141**	EM	2295293	1	60	58	1.67	20.80	12	4	2	4	60	60	0	100	75	95	6	3	3	7	6	7	0	4.85	1	0.00	0	0	4.85
**142**	ENM	2311579	1	61	62.5	1.80	19.29	13	2	1	5	60	60	0	95	30	65	4	2	1	9	3	9	0	3.00	0	0.00	0	0	3.00
**143**	control	2310911	1	47	80	1.70	27.68	15	1	3	2	60	60	0	100	50	55	5	2	2	6	7	7	1	9.68	1	228.63	1	0	9.68
**144**	EM	2169744	0	66	60	1.50	26.67	16	3	7	1	60	60	0	100	65	65	6	3	3	7	3	7	0	3.01	1	0.00	0	0	3.01
**145**	EM	2311807	0	65	52	1.60	20.31	9	1	3	2	60	60	0	100	75	85	2	2	1	3	2	3	0	5.48	0	0.00	0	0	5.48
**146**	control	21029854	1	89	65	1.74	21.47	6	1	5	2	60	54	0	90	60	10	3	1	2	3	5	5	2	9.37	1	0.00	0	0	9.37
**147**	control	2283382	0	65	60	1.56	24.65	16	4	7	3	60	60	0	100	55	70	3	1	2	4	4	4	0	3.03	1	0.00	0	0	3.03
**148**	ENM	2117547	0	74	55	1.65	20.20	18	2	5	2	58	60	0	95	30	65	5	2	2	5	3	5	0	6.88	1	165.05	1	0	6.88
**149**	EM	2238236	0	54	60	1.60	23.44	8	1	7	4	60	60	0	95	70	85	0	1	1	2	3	3	1	3.00	1	0.00	0	0	3.00
**150**	EM	2318516	0	63	60	1.50	26.67	12	1	7	4	60	54	0	100	45	5	6	3	3	10	13	14	4	6.16	0	0.00	0	0	6.16
